# A Synergistic Transcriptional Regulation of Olfactory Genes Drives Blood-Feeding Associated Complex Behavioral Responses in the Mosquito *Anopheles culicifacies*

**DOI:** 10.3389/fphys.2018.00577

**Published:** 2018-05-23

**Authors:** Tanwee Das De, Tina Thomas, Sonia Verma, Deepak Singla, Charu Chauhan, Vartika Srivastava, Punita Sharma, Seena Kumari, Sanjay Tevatiya, Jyoti Rani, Yasha Hasija, Kailash C. Pandey, Rajnikant Dixit

**Affiliations:** ^1^Laboratory of Host-Parasite Interaction Studies, National Institute of Malaria Research, Dwarka, India; ^2^Department of Biotechnology, Delhi Technological University, Rohini, India; ^3^Department of Biochemistry, National Institute for Research in Environmental Health, Indian Council of Medical Research, Bhopal, India

**Keywords:** mosquito, host-seeking, blood feeding, behavior, olfaction

## Abstract

Decoding the molecular basis of host seeking and blood feeding behavioral evolution/adaptation in the adult female mosquitoes may provide an opportunity to design new molecular strategy to disrupt human-mosquito interactions. Although there is a great progress in the field of mosquito olfaction and chemo-detection, little is known about the sex-specific evolution of the specialized olfactory system of adult female mosquitoes that enables them to drive and manage the complex blood-feeding associated behavioral responses. A comprehensive RNA-Seq analysis of prior and post blood meal olfactory system of *An. culicifacies* mosquito revealed a minor but unique change in the nature and regulation of key olfactory genes that may play a pivotal role in managing diverse behavioral responses. Based on age-dependent transcriptional profiling, we further demonstrated that adult female mosquito's chemosensory system gradually learned and matured to drive the host-seeking and blood feeding behavior at the age of 5–6 days. A time scale expression analysis of Odorant Binding Proteins (OBPs) unravels unique association with a late evening to midnight peak biting time. Blood meal-induced switching of unique sets of OBP genes and Odorant Receptors (Ors) expression coincides with the change in the innate physiological status of the mosquitoes. Blood meal follows up experiments further provide enough evidence that how a synergistic and concurrent action of OBPs-Ors may drive “prior and post blood meal” associated complex behavioral events. A dominant expression of two sensory appendages proteins (SAP-1 & SAP2) in the legs of *An. culicifacies* suggests that this mosquito species may draw an extra advantage of having more sensitive appendages than *An. stephensi*, an urban malarial vector in the Indian subcontinents. Finally, our molecular modeling analysis predicts crucial amino acid residues for future functional characterization of the sensory appendages proteins which may play a central role in regulating multiple behaviors of *An. culicifacies* mosquito.

**SIGNIFICANCE**

Evolution and adaptation of blood feeding behavior not only favored the reproductive success of adult female mosquitoes but also make them important disease-transmitting vectors. An environmental exposure after emergence may favor the broadly tuned olfactory system of mosquitoes to drive complex behavioral responses. But, how these olfactory derived genetic factors manage female specific “pre and post” blood meal associated complex behavioral responses are not well known. Our findings suggest that a synergistic action of olfactory factors may govern an innate to prime learning strategy to facilitate rapid blood meal acquisition and downstream behavioral activities. A species-specific transcriptional profiling and an *in-silico* analysis predict that “sensory appendages protein” may be a unique target to design disorientation strategy against the mosquito *Anopheles culicifacies*.

## Introduction

Mosquitoes are one of the deadliest living animals, transmitting a variety of infectious diseases such as malaria, dengue fever, chikungunya and zika fever worldwide. According to WHO report, malaria is one of the major vector-borne diseases that causes 212 million morbidity cases and more than 4 million mortalities (World Health Organization, 2015). WHO recognized that in India, malaria situation is more complex and puts an estimated socio-economic burden of $1.94 billion annually (World Health Organization, 2015). Current tools to control and manage malaria face challenges due to the emergence of drug resistance in parasite and insecticide resistance in mosquitoes (Stein et al., 2009; Petersen et al., 2011; Winzeler and Manary, 2014; Cui et al., 2015; Liu, 2015; Sahu et al., 2015). Thus, alternative molecular tools are required to rule out the expanding vector population as well as parasite development.

One of the key molecular strategies under not-to-bite approach relies on the designing of a new class of molecular tools that are able to disorient/alter the adult female mosquitoes host-seeking behavior (Potter, 2014). Therefore, defining the molecular basis of host-seeking behavioral evolution and adaption to blood feeding by the adult female mosquitoes remains central to our understanding. This may probably be due to the complex interaction of genetic and non-genetic factors, driving mosquito navigation (Takken and Verhulst, 2013). In nature mosquitoes encounter many challenges to sustain in daily life viz. they rely immensely on their sense of smell (olfaction) for the majority of their lifecycle stages (Potter, 2014). The well-developed nasal system of mosquitoes is able to detect and discriminate thousands of different odor molecules and thus play an essential role in the facilitation of olfactory guided behavior. These complex behavioral events are largely mediated by the diverse chemosensory genes encoding odorant binding proteins (OBPs), odorant degrading enzymes (ODEs), odorant receptors (Ors) and other accessory proteins including sensory neuron membrane protein (SNMP) (Takken and Knols, 2010). Odorant binding proteins (OBPs), which are bathed within the sensillum lymph, are low molecular weight soluble proteins that mediate the first interaction of the olfactory system with the external world (Takken and Knols, 2010; Carey and Carlson, 2011; Brito et al., 2016). These globular protein molecules showed significant diversity within the same family and are believed to bind with a wide range of hydrophobic odorant molecules. After binding with the odor molecules, OBPs transport it to their respective olfactory receptors present on the olfactory receptor neurons (ORNs) (Takken and Knols, 2010; Fan et al., 2011; Martin et al., 2011). Olfactory receptors (OrX) of insects' are associated with the obligate receptor co-receptor (Orco) on the dendritic membrane of ORN for proper functioning (Takken and Knols, 2010). Orco is not only essential for dendritic trafficking and presentation of the OrX in the membrane but also facilitate the formation of odorant gated ion channels by structural alteration that is opened upon odorant binding (Zwiebel and Takken, 2004; Takken and Knols, 2010).

The genome sequencing of several *Anopheline* sp. facilitates the identification of different olfactory genes including OBPs and Ors from different mosquito species. Functional characterization of few *Anopheline* mosquitoes OBP genes (OBP1, OBP20, OBP7, OBP2, OBP48) highlights their role in host-seeking behavioral activities (Biessmann et al., 2005, 2010; Li et al., 2005; Sengul and Tu, 2008, 2010; Hoffman et al, 2012; Tsitsanou et al., 2013; Ziemba et al., 2013). Consequently, de-orphanization of several odorant receptors (AgOr1, AgOr2, AgOr8, AgOr5, AgOr65) from *An. gambiae* also showed their specificity to human-specific odorant molecules (Hallem et al., 2004; Carey et al., 2010). After binding of the odorant molecules with their cognate receptors, the actual signal transduction cascade is initiated which involves either the activation of ligand-gated ion channels or stimulation of the secondary messenger pathway (Takken and Knols, 2010). In insects, including mosquitoes, a combinatorial coding mechanism of the olfactory system is believed to increase the sensitivity of the odorant reception, which enables them to respond to specific odorants (Martin et al., 2011; Andersson et al., 2015). Thus, it is plausible to hypothesize that prior blood meal, key interactions of odorants and their cognate receptors may have a significant influence on food choice decision and blood meal uptake process.

For a successful blood feeding event, an adult female mosquito needs to manage multiple behavioral coordinates including searching, locating, landing over a suitable host, followed by tracing the proper site to pierce and suck the blood within 2 min (Zwiebel and Takken, 2004; Benoit et al., 2011; Sim et al., 2012; McMeniman et al., 2014; Cardé, 2015; Van Breugel et al., 2015; Won Jung et al., 2015). Just after the piercing organ (proboscis), it is the salivary gland which mediates the immediate biochemical interaction with the vertebrate blood and facilitate rapid blood meal uptake. Our recent study suggested that adult female mosquito's salivary glands are evolved with the unique ability of gene expression switching to manage meal specific (sugar vs. blood) responses (Sharma et al., 2015b), but the molecular nature of the olfactory and neuro-system in regulating the salivary gland function is yet to unravel.

Mosquitoes after taking a blood meal, need to enter into a new habitat favoring successful oviposition (Rinker et al., 2013; Day, 2016). In fact, after blood meal acquisition, mosquitoes undergo two major behavioral switching events; (i) searching for suitable site(s) for temporary resting and completion of blood meal digestion (~30 h) which is necessary for egg maturation (48–72 h); and (ii) finding a proper oviposition site for successful egg laying (Taparia et al., 2017). After completion of egg laying event, the adult female mosquitoes regain their host-seeking activity for a second blood meal to complete the next gonotrophic cycle (Takken et al., 2001; Rinker et al., 2013). Notably, “prior and post” blood meal associated habitats may have a significant difference in their physical, chemical and biological characteristics (Day, 2016), but the molecular basis that how olfactory-driven factors manage these complex events is still not well understood (Chen et al., 2017).

Immediately after mosquito emergence, an exposure to diverse environmental/chemical cues facilitate the maturation and learning of the olfactory machinery components (sensory appendages, maxillary palps and proboscis) to govern common innate behavioral activities such as nectar sugar feeding and mating in both the sexes (Takken and Verhulst, 2013; Lutz et al., 2017). However, it is yet not clear whether the mating events have any direct impact on the initiation of host seeking and blood feeding behavioral responses. Our recent finding suggested that *quick-to-court* protein may have a crucial role to meet the conflicting demand of sexual mate partner finding and/or a suitable vertebrate host finding by regulating the expression of unknown olfactory genes in adult *An. culicifacies* mosquito (De et al., 2017). In fact, the organization of the olfactory components is morphologically similar in both the sexes but carries unique structural differences which are responsible for discrete temporal peaks of activities to sense swarm and identify sex partner for a successful mating event (Pitts et al., 2011). However, in case of adult female mosquitoes, we opined that the evolutionary forces might have driven an extra specialization of the olfactory components such as proboscis, enabling rapid host seeking and blood feeding behavioral adaptation. In other words, we termed this highly sex-specific extra specialization as an “evolutionary speciality” which not only evolve adult female mosquitoes as a fast blood feeder but make them a potent vector for many disease pathogens. Once, a mosquito takes first blood meal it needs to manage major physiological activities linked to blood meal digestion and egg maturation. These physiological changes possibly may have another level of impact on olfactory perception to guide oviposition site finding behavior. We further hypothesize that first blood meal exposure must have a priming effect on the olfactory responses expediting the consecutive host seeking and blood feeding behavioral activities more rapidly than previous one.

To test and decode this evolutionary speciality, we performed RNA-Seq analysis of the complete olfactory system of adult female *An. culicifacies* mosquito, a dominant Indian malarial vector. A comprehensive molecular and functional annotation of RNA-Seq data unraveled a limited but remarkable change in the nature and regulation of unique sets of olfactory gene repertoire in response to distinct feeding status of the mosquitoes. Extensive transcriptional profiling of the selected transcripts showed biphasic and synergistic regulation under the distinct innate physiological status of the mosquitoes, possibly to facilitate and manage the complex host-seeking behavioral events. Finally, our structural bioinformatic analysis predicts the key residues of the selected sensory appendages proteins for future functional validation and characterization as a unique target to design disorientation strategy against the mosquito *An. culicifacies*, responsible for more than 65% malaria cases in India (Sharma and Dev, 2015).

## Materials and methods

**Figure 1:**
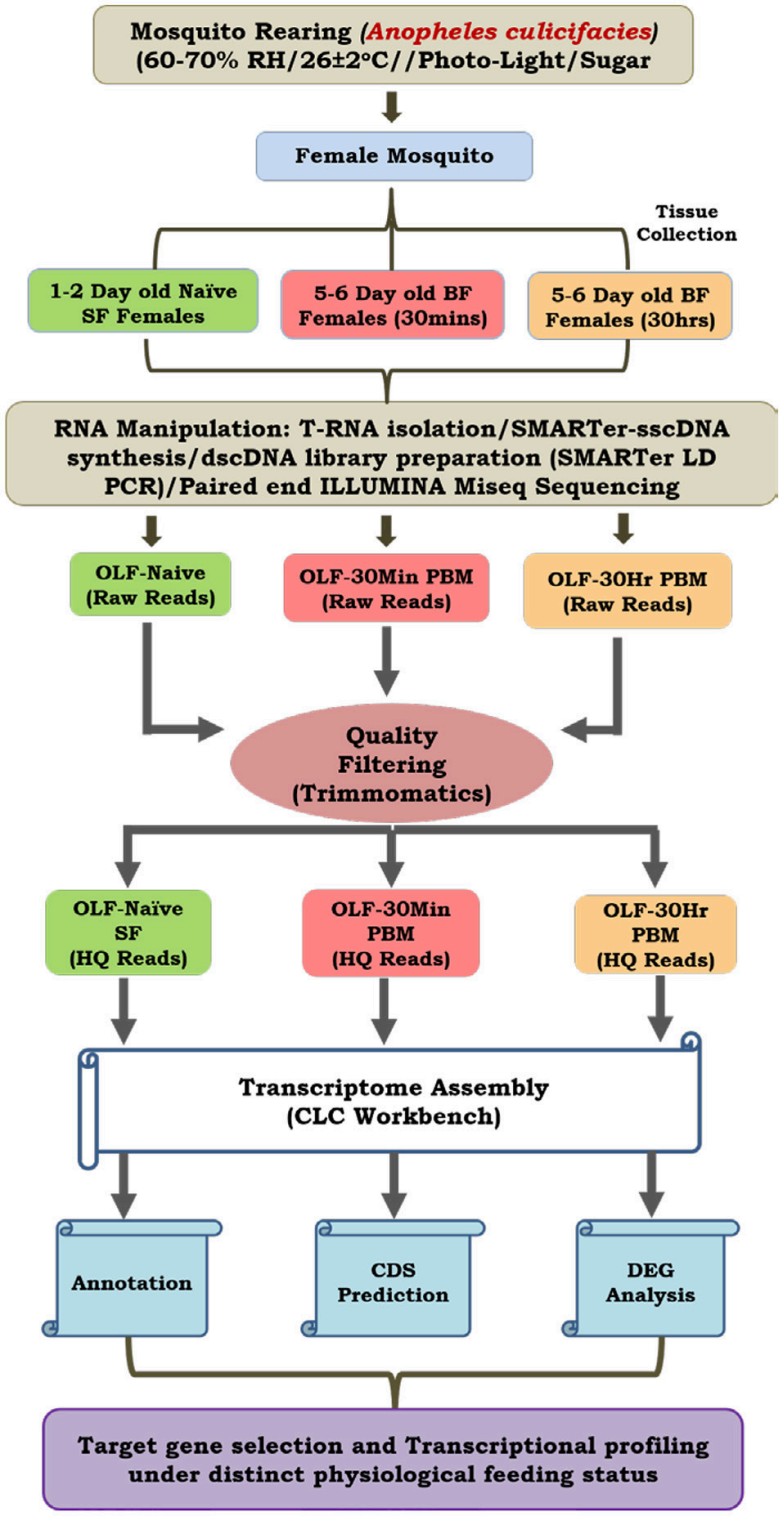
A technical overview to decode the hard-wired genetic structure of the olfactory system of *Anopheles culicifacies*. We sequenced and analyzed three RNA samples extracted from the olfactory tissues of approximately 30 mosquitoes individually and pooled to form one single sample. With this strategy, we were able to quantify and validate the estimation of gene expression, expected with a minimum chance of aberrations as described earlier (Sharma et al., 2015b).

Figure 1 represents a technical overview of the current investigation.

### Mosquito rearing and maintenance

A cyclic colony of the mosquito *An. culicifacies*, sibling species A and *An. stephensi* were reared and maintained at 28 ± 2°C, RH = 80% in the central insectary facility as mentioned previously (Thomas et al., 2014; Sharma et al., 2015b). All protocols for rearing and maintenance of the mosquito culture were approved by ethical committee of the institute.

### RNA isolation and transcriptome sequencing analysis

Complete olfactory tissue which includes antennae, maxillary palp, proboscis and labium, were dissected from 0 to 1 day of age, 30 min post blood fed and 30 h post blood fed *An. culicifacies* mosquito and collected in Trizol Reagent. Total RNA isolated from the collected olfactory tissues of approximately 30 mosquitoes was pooled to form one single sample and a double-stranded cDNA library for each set of naïve, 30 min and 30 h post blood meal, was prepared by a well-established PCR-based protocol described previously (Dixit et al., 2011; Sharma et al., 2015b). Whole transcriptome sequencing of the olfactory tissue was performed using the Illumina MiSeq 2 X 150 paired-end library preparation protocol. The sequencing data analysis pipeline is shown in Figure 1. Briefly, raw reads from each set were processed for removing the adaptors and low-quality bases (<20). A denovo clustering using CLC Genomics Workbench (V6.2) (Zhu et al., 2014) was used to build final contigs/transcripts dataset with default parameters (contig length ≥ 200, Automatic word size: Yes, Perform Scaffolding: Yes, Mismatch cost: 2, Inserstion cost: 3, Deletion cost: 3, length fraction: 0.5, Similarity fraction: 0.8). Finally, assembled transcriptome was used for CDS prediction and annotation using transdecoder and BLASTX at *e*-value 1e^−6^, respectively.

For a comprehensive differential gene expression (DGE) analysis we used DESeq R Package as described earlier (Chen et al., 2017). Briefly, the high quality reads for each sample were mapped on their respective set of CDS/transcripts and FPKM (Fragments Per Kilobase of Exon Per Million Fragments Mapped) values were calculated using following formula i.e., FPKM = 10^∧^9 x C / (N x L), where C is the number of reads mapped onto the CDS; N the total number of mapped reads in the experiment; and L is the number of base pairs in the CDS. The common hit accessions based on BLAST against NR database were identified for differential gene expression analysis. CDS were further classified as up and down-regulated based on their log fold change (FC) value, which was calculated by the using the formula: FC = Log_2_ (Treated/Control). Because, DESeq calculates raw *p*-values using a negative binomial distribution accounting technical and biological variables, and later *p*-values are corrected for multiple testing using the Benjamini-Hochberg statistical procedure which controls false discovery rate (FDR). Transcripts pairs whose read numbers displayed a greater than two-fold difference with *P* < 0.05 was listed as differentially expressed genes.

### Identification and molecular cataloging of olfactory genes in *An. culicifacies*

An initial BLAST search analysis predicted a total of 93 transcripts encoding putative OBP homologs from the olfactory transcriptome data of *An. culicifacies* mosquito. To predict additional OBPs, a merged OBPs database of mosquito and *Drosophila* was re-queried against *An. culicifacies* draft genome/predicted transcripts databases available at www.vectorbase.org and build up the final OBP catalog for phylogenetic analysis as detailed in the Figure S1. A PDB database homology search analysis and GO annotation was used to identify and catalog other putative olfactory receptor genes manually.

### PCR based gene expression analysis

The head tissue containing the olfactory appendages of female *An. culicifacies* mosquito was dissected at different zeitgeber time point. The 24 h time scale of the LD cycle is represented as different Zeitgeber time (ZT) where ZT0 indicate the end of dawn transition, ZT11 is defined as the start of the dusk transition and ZT12 is defined as the time of lights off (Rund et al., 2013). At the same time other tissues such as. head (male, female), legs (male, female), brain, olfactory tissue (OLF), female reproductive organ (FRO) and male reproductive organ (MRO) of both *An. culicifacies* and *An. stephensi* mosquitoes were also dissected and collected in Trizol followed by total RNA extraction and cDNA preparation. Differential gene expression analysis was performed using the normal RT-PCR and agarose gel electrophoresis protocol. For relative gene expression analysis, SYBR green qPCR (Thermo Scientific) master mix and Illumina Eco Real-Time PCR machine were used. PCR cycle parameters involved an initial denaturation at 95°C for 5 min, 40 cycles of 10 s at 95°C, 15 s at 52°C, and 22 s at 72°C. Fluorescence readings were taken at 72°C after each cycle. The final steps of PCR at 95°C for 15 s followed by 55°C for 15 s and again 95°C for 15 s were completed before deriving a melting curve. Each experiment was performed in three independent biological replicates to better evaluate the relative expression. Actin or S7 gene was used as internal control in all the experiment and the relative quantification was analyzed by 2^−ΔΔ*Ct*^ method (Livak and Schmittgen, 2001). Differential gene expression was statistically analyzed using student *t*-test.

### Blood meal time series follow up

Figure S9 represents a technical overview of the blood meal follow up experimental protocol. Briefly, the olfactory tissues were collected from 25 to 30 adult female mosquitoes for both naïve sugar-fed and blood fed mosquitoes at different time points. Olfactory tissues collections were initiated from 0 to 1 day of naïve sugar-fed mosquitoes and proceed up to 6–7 days on every alternative day. After the 6th day, the adult female mosquitoes were offered first blood meal by offering a live animal (rabbit) and immediately collected olfactory tissues for 30 min time point. The full blood-fed mosquitoes were separated and kept in a proper insectary condition for further experiment. After collection of olfactory tissues at 30 h and 72 h post blood fed the gravid females were kept for oviposition and again dissected OLF tissues after 24 h of the egg laying event. Second blood meal was provided to the egg laid mosquitoes and final collection of OLF tissues was done after 30 h of 2^nd^ blood meal. Initially, relative expression data were interpreted to evaluate a general response using one way analysis of variance (ANOVA) for multiple comparison, however, wherever required “test” sample data was compared with “control” data set and statistically analyzed using Student's *t*-test.

### Structural modeling of SAP1 and SAP2

The structure prediction analysis of SAP1 and SAP2 proteins from *An. culicifacies* was carried out through searching of a template for each query proteins against PDB database using BLASTP algorithm. Based on highest query coverage, identity and *e*-value, two best templates were selected for each used query sequence and thereafter, modeller9 v.13 was used for the building of 50 models for each query sequence using multiple templates. The best model was selected using DOPE (Discrete Optimized Protein Energy) score, which is favored by the lowest cumulative energy score for the whole structured model. The selected model was further validated by Ramachandran plot using PROCHECK software which estimates the stereo-chemical quality of the residues in allowed, disallowed and favorable regions. Finally, the selected models were used for binding site prediction using COACH software.

## Results

### Blood meal causes modest but unique changes to olfactory responses

**Figure 2:**
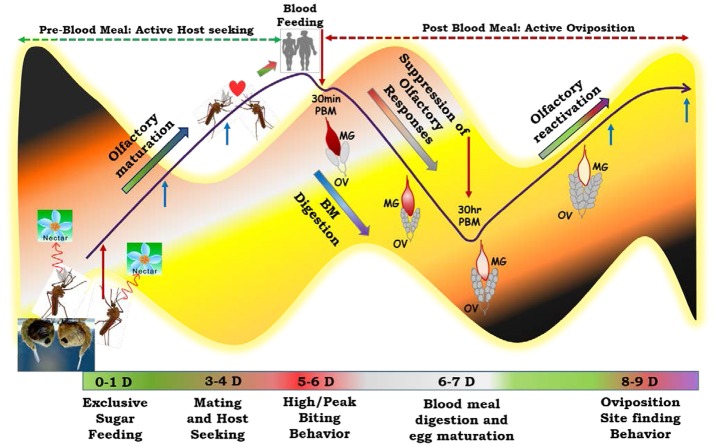
Working Hypothesis to establish functional co-relation of the olfactory system under distinct feeding status: Adult mosquitoes, just after emergence from pupae are exclusive sugar feeders and dependent on nectar sugar to acquire energy for flight activity. Exposure of the adult mosquitoes to the diverse aromatic environment facilitates their learning and maturation of the olfactory system which enables successful mating and host-seeking behavioral activities. But the function of the olfactory system starts to diminish just after blood feeding and become ceased at least for 30 h of post blood meal. Blood feeding initiates lots of physiological changes including blood meal digestion in the midgut and egg maturation in the ovary which consume lots of energy and thus mosquitoes manipulate the energy cost by shutting down the olfactory responses and preferred to take rest at a cool dark place. After 30 h of blood feeding the blood almost digested in the midgut and maturation of egg reached a threshold level which reinforces the mosquito to perform to next level of behavior. Thus, recovery/reactivation of the olfactory responses occurs to find a suitable site for egg laying/oviposition. To capture this molecular snapshot and track the events, we collected olfactory tissues at three different physiological conditions for RNA-Seq analysis (Highlighted as red arrows) and coupled with gene expression study with more elaborated time and physiological state (highlighted with blue arrows). MG, Midgut; OV, Ovary. Mosquitoes each and every life cycle stages are tightly regulated by circadian (dawn & dusk) cycle (Background light dark color code).

To decode and establish the possible molecular relationship managing “prior and post” blood meal behavioral events we developed a working hypothesis (Figure 2), a plausible mechanism which may have a significant influence on mosquito feeding and survival in diverse ecologies. To test this hypothesis, first we generated and analyzed a total of ~122 million RNA-Seq reads of the olfactory tissues collected from 1 to 2 day old naive (Nv), 5- 6-day old immediate blood fed (30 m-2 h PBM) and 30 h post blood fed (30 h PBM) mosquitoes (Table 1). We chose 30 h PBM as a critical time when completion of blood meal digestion occurs in the midgut, which may have a direct influence on the reactivation of the olfactory system (Figure S2) (Gonalves et al., 2009; Rinker et al., 2013; Taparia et al., 2017). For molecular and functional annotation, we assembled each transcriptomic database into contigs/transcripts and compared against multiple molecular databases as described earlier (Sharma et al., 2015b). Supplementary Table 1 represents details of the annotation kinetics of mosquito olfactory databases.

**Table 1:** Catalogue of Odorant Binding Proteins of *An. culicifacies*.

**Sl no**.	**Sample name**	**Number of OBPs transcripts**
**(A)**		
1.	Ac-OLF-Naïve	14
2.	Ac-OLF-30 min PBM	10
3.	Ac-OLF-30 h PBM	12
4.	Ac-genome retrieved	27
	Total OBPs in *An. culicifacies*	63
**Sl no**.	**Family of OBPs**	**Number**
**(B)**		
1.	Classic OBPs	26
2.	Plus-C OBPs	13
3.	Two-domain OBPs	13
4.	Other chemosensory proteins	11

**Figure 3:**
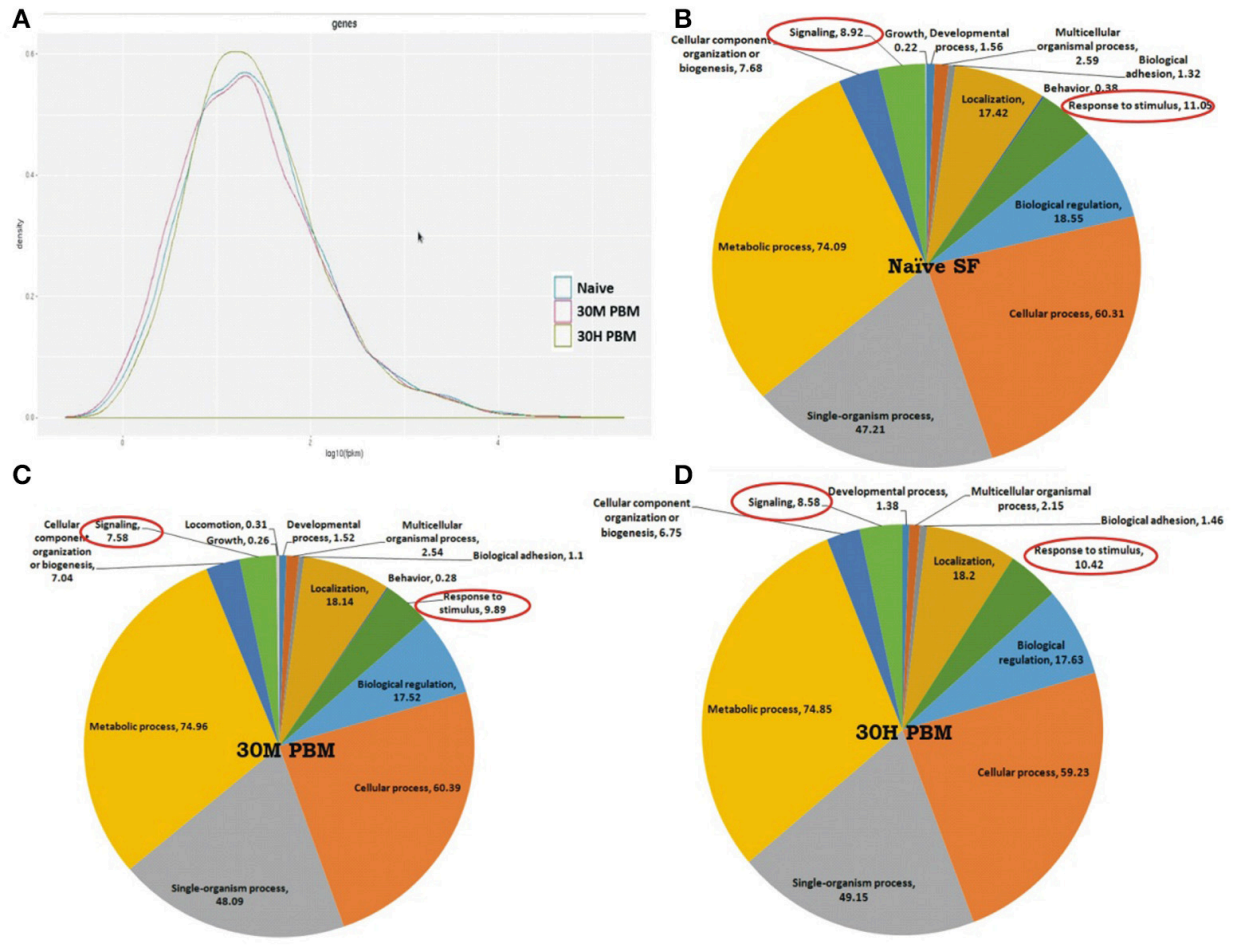
Blood meal cause modest changes in the molecular architecture of the mosquito olfactory system**: (A)** Read density map of the compared Naive; 30 m and 30 h post blood meal (PBM) transcriptomic data of olfactory system; **(B–D)** Functional annotation and molecular catalog of olfactory transcriptome (Biological Process/Level2/% Transcripts). The red circle marks the genes selected for transcriptional response monitoring. (See Text).

To test whether blood meal alters the global expression pattern of the olfactory transcriptome, we performed a differential gene expression analysis. Initial attempt of mapping cleaned reads to the available draft reference genome failed to yield quality results, probably due to poor annotation (Figure S3). Alternatively, we mapped all the high quality reads against *denovo* assembled reference map, as described earlier (Sharma et al., 2015b). Blood meal causes a modest shift in the transcriptome expression (Figure 3A), supporting the previous report that first blood meal enhances odorant receptor transcripts abundance modestly, but causes general reduction of mosquito antennal chemosensory gene repertoire in *An. gambiae* (Rinker et al., 2013).

We observed that at least 85% transcriptome remains unaltered, while only ~6% transcripts are up-regulated and ~8.7% transcripts downregulated in 30 min post blood fed samples (Supplementary Table 2 and Dataset S1). As expected, ~10% transcripts expression was further reduced in 30h post blood fed olfactory tissue samples while only 2% transcripts were up-regulated when compared to naive sugar-fed mosquitoes (Supplementary Table 2). Interestingly, a comprehensive annotation analysis also predicted that basic composition of the mosquito olfactory tissue does not alter significantly (Figures 3B–D). This observation allowed us to further hypothesize that blood-feeding may not directly cause a major shift in transcript abundance but may alter the functional nature/regulation of the unique transcripts controlling key biological processes such as response to stimulus, circadian rhythm and signaling in the blood fed adult female mosquitoes (Figures 3B–D). To clarify this complexity, we manually shortlisted the olfactory transcripts either based on their FPKM abundance and/or predicted coding nature and analyzed a set of unique genes likely to influence mosquito host-seeking and blood-feeding behavior. To trace the possible molecular link, we extensively profiled their transcriptional regulation under distinct feeding status (see below).

### Daily rhythm and expression change of odorant binding proteins (OBPs) may influence olfactory responses

To negotiate and manage the navigation trajectory toward the vertebrate host, olfactory encoded odorant binding proteins (OBPs) play a crucial role to bind and deliver the odorants/chemicals to their cognate odorant receptors, an event guiding behavioral decisions. To explore the possible role of OBPs in the regulation of the olfactory behavior we identified and cataloged a total of sixty-three OBP genes by homology search analysis from the mosquito *An. culicifacies* (Table 1A). Domain prediction analysis classified the OBPs as Classic OBPs, Plus-C OBPs, Two-domain OBPs and other Chemosensory protein family (Table 1B; details in Supplementary Table 3), as described earlier for the mosquito *An. gambiae* (Manoharan et al., 2013).

A comprehensive phylogenomic analysis of the Classic putative OBPs of *An. culicifacies* highlights the conserved sequence relationship with *An. gambiae* and other mosquito/insect species (Figure S4A). Whereas, Plus-C OBPs and more dominantly Atypical OBPs seem to be unique to the mosquitoes suggesting their possible involvement in the evolution and adaptation of blood feeding behavior of adult female mosquitoes (Figures S4B,C).

Interestingly, differential gene expression (DGE) data indicated that blood meal restricted the expression of common OBP transcripts (Figure S5). However, first blood meal causes the appearance of unique OBP transcripts (Table 1), a crucial event in modulating the behavioral activities in response to change in the feeding status i.e., naive sugar to blood feeding. To further validate and unravel this unique relationship of OBPs regulation, we examined the RT-PCR based expression of at least 11 putative OBP transcripts under distinct feeding status of the mosquitoes. In this analysis, we also included two chemosensory proteins (CSPs) named sensory appendage protein (SAP1 & SAP2) having a dominant expression in the naive mosquito olfactory tissue (Supplementary Table 3).

**Figure 4:**
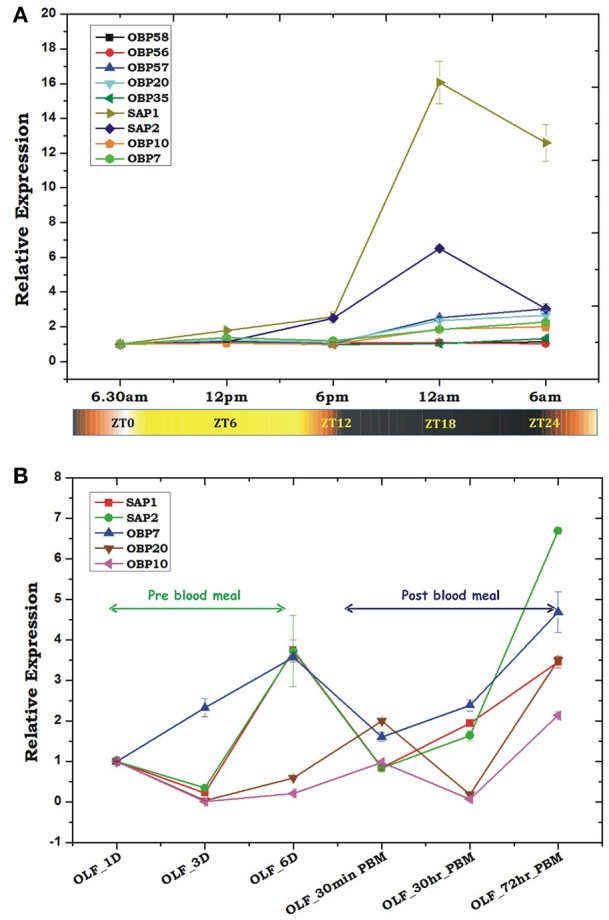
Transcriptional profiling of the odorant-binding protein genes (OBPs) under different circumstances. **(A)** Rhythmic expression of OBP genes in the adult female's olfactory tissues (OLF) according to different zeitgeber time (ZT) scale, where ZT0 indicate the end of dawn transition, ZT11 is defined as the start of the dusk transition and ZT12 is defined as the time of lights off. **(B)** Relative expression profiling of OBP genes in pre and post blood fed olfactory tissues. Olfactory tissues (OLF) were collected from 1, 3, and 6 day old sugar-fed mosquitoes which were then provided blood meal and then the olfactory tissues were collected after 30 min of post blood fed and 30 and 72 h of post blood-fed mosquitoes. The significance of suppression of OBP genes expression after 30 h of post blood meal are as follows: SAP ≤ 0.004; SAP2 ≤ 0.039; OBP7 ≤ 0.007; OBP20 ≤ 0.0004; OBP10 ≤ 0.003.

Our Zeitgeber time scale expression showed that out of tested nine OBPs transcripts, at least 6 OBP transcripts showed a >2-fold modulation in their expression during late evening to midnight, in the 6-day old naïve mosquitoes (Figure 4A). These data also corroborate with the previous observation that the natural active biting behavior of *An. culicifacies* mosquito occurs in the mid-night (Singh et al., 1995; Basseri et al., 2012). Surprisingly, sensory appendage proteins (Ac-SAP1 & Ac-SAP2) showed unequivocally an enriched (16-fold for SAP1, *p* ≤ 0.001 and 6-fold SAP2, *p* ≤ 0.0001) expression than other tested OBPs. Apparently, we also observed a transient suppression (30 min) and rapid recovery of OBPs expression just after a first blood meal (Figure 4B). However, surprisingly, OBP7 showed a unique pattern of a consistent up-regulation till the 6th day when compared to a gradual enrichment of other tested OBP and SAP after 3-day post-emergence in the naive adult female mosquitoes. However, it is yet to be clarified whether an early enrichment of OBP7 has any important role in aging mosquitoes' olfactory responses.

### Innate physiological status may influence olfactory receptor responses to manage behavioral switching events

**Figure 5:**
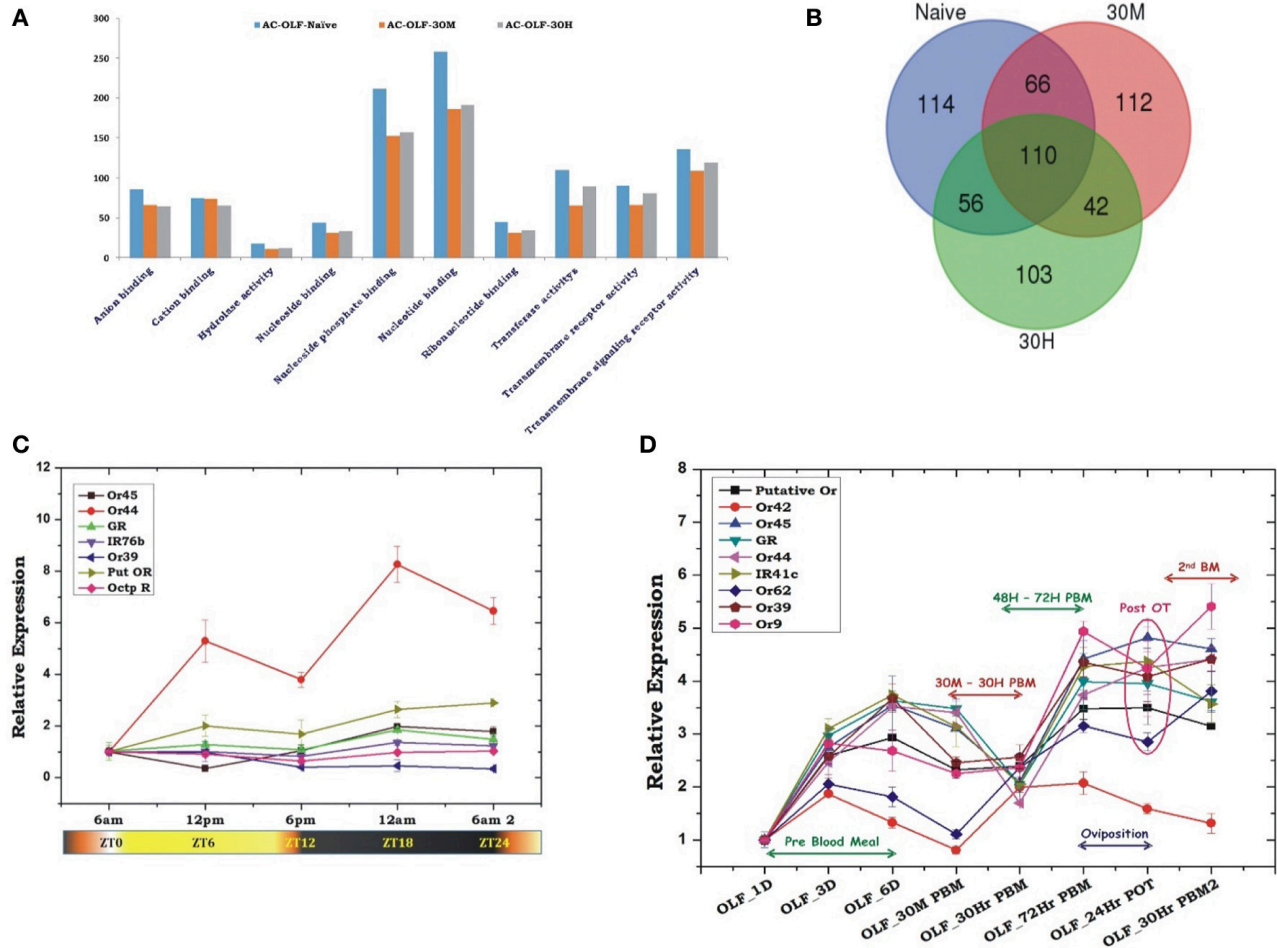
Blood meal modulates odorant receptors expression. **(A)** A comparative GO score distribution analysis of the response to stimulus and signaling transcripts of naïve and blood-fed mosquitoes. **(B)** Venn diagram showing common and unique transcripts of response to stimulus and signaling GO category of naïve and blood-fed mosquitoes. **(C)** Rhythmic expression of olfactory receptor genes (Ors) of *An. culicifacies* in the olfactory tissues of female mosquitoes, where ZT0 indicate the end of dawn transition, ZT11 is defined as the start of the dusk transition and ZT12 is defined as the time of lights off. **(D)** Transcriptional response of olfactory receptor genes according to blood meal time series experiment. Olfactory tissues (OLF) were collected from naïve sugar fed adult female mosquitoes till 6th day (OLF-1D, OLF-3D, OLF-6D). Then mosquitoes were provided blood meal and again olfactory tissues were collected at a different time point after blood feeding, viz. OLF-30 M: 30 min post blood fed (PBM); 30 h-PBM: 30 h of PBM; 72 h-PBM: 72 h of post blood meal; then the mosquitoes were kept for oviposition (egg laying), and again the olfactory tissues were collected 24 h of post oviposition (24 h-POT). Finally, the 2nd blood meals were provided to the egg laid mosquitoes and collected olfactory tissue 30 h of second blood meal (30 h-PBM2). A multiple comparison analysis (ANOVA) revealed a significant modulation in the expression of each gene (mean significant *p* < 0.05). Further, the significance of suppression of OR genes expression after 30 h of post blood meal are as follows: Putative Or ≤ 0.001; Or42 ≤ 0.05; GR ≤ 0.003; Or44 ≤ 0.0002; IR41c ≤ 3.5E^−05^; Or62 ≤ 0.06; Or39 ≤ 0.02; Or9 NS.

A transient modulation of OBPs expression in response to blood meal further prompted us to decode and establish its correlation with the olfactory receptors. To unravel this relationship, initially we retrieved, pooled and cataloged a total of 603 unique transcripts linked to response to stimulus and signaling (RTSS) categories (Figures 3B–D), encoding diverse nature of proteins such as anion binding, nucleic acid binding, receptor activity, hydrolases and transferase activity (Figure 5A). A comparative GO score distribution analysis predicted lower score hits for the blood-fed cohorts than naive mosquitoes (Figure 5A). Surprisingly, out of 603 transcripts, we noticed only 110 transcripts were common to all, while >100 transcripts remain uniquely associated with individual physiological conditions compared in the study (Figure 5B).

Olfactory receptors play a central role to receive and communicate the initial chemical message to the higher brain center through ORNs for decision-making events. Thus, we made a catalog of 50 different chemosensory receptors (Table 2), comprising odorant receptors (Ors); gustatory receptors (Grs) and variant ionotropic receptors (Irs), which appeared predominantly in the naïve and blood fed cohorts of the RTSS category (Supplementary Table 4). Interestingly, a cluster of 19 different olfactory receptor genes was found to be expressed abundantly and exclusively in the naïve mosquito (Supplementary Table 4). At the same time, we also observed that a distinct repertoire of chemosensory receptor genes uniquely appeared in the blood fed cohorts, but their number is much lower than the naïve mosquito (Supplementary Table 4). Observation of the constitutive expression of Orco and few other Ors and Grs (totaling 10 transcripts) in all the experimental conditions highlighted the importance of Orco for the presentation of other receptors in the olfactory system.

**Table 2:** Number of Odorant Receptors retrieved from the olfactory tissue of naive and blood fed *An. culicifacies* mosquito.

**Sl. no**.	**Sample name**	**Number of olfactory receptors transcripts**
1.	Ac-OLF-naive	32
2.	Ac-OLF-30 min PBM	11
3.	Ac-OLF-30 h-PBM	7
4.	Total	50

Unlike OBPs, poor modulation of olfactory receptor gene expression under circadian rhythm (Figure 5C) suggested their minimal role in the initialization of host-seeking behavioral activities. Alternatively, we also interpreted that Ors may not have direct biphasic regulation, but may influence a successful blood feeding event. To further corroborate with the above propositions and uncover the functional correlations of olfactory receptor responses, we monitored the transcriptional regulation of the selected Or transcripts in response to two consecutive blood meal series follow-up experiment. An age-dependent enrichment of Or transcripts till 6th day of maturation in the sugar-fed mosquitoes suggested that naïve mosquitoes may express and attain a full spectrum of chemosensory genes expression to meet all the needs of their life cycle requirements i.e., host-seeking and mate-finding behavioral response (Figure 5D).

First blood meal to the 6^th^-day old naïve mosquitoes initiates the suppression of almost all the olfactory receptor transcripts within 30 min of blood feeding, whose expression almost ceased to a basal level at 30 h post blood meal, except the slight up-regulation of two transcripts named Or42 and Or62 (Figure 5D). Apparently after 30 h PBM, we observed a significant modulation of the receptor gene expression which started enriching till 72 h of post first blood meal, a time window coincides with the successful completion of the oviposition event. However, we did not observe any significant change in the expression of the receptor transcripts in response to second blood meal (Figure 5D).

### Blood meal response to other olfactory proteins

**Figure 6:**
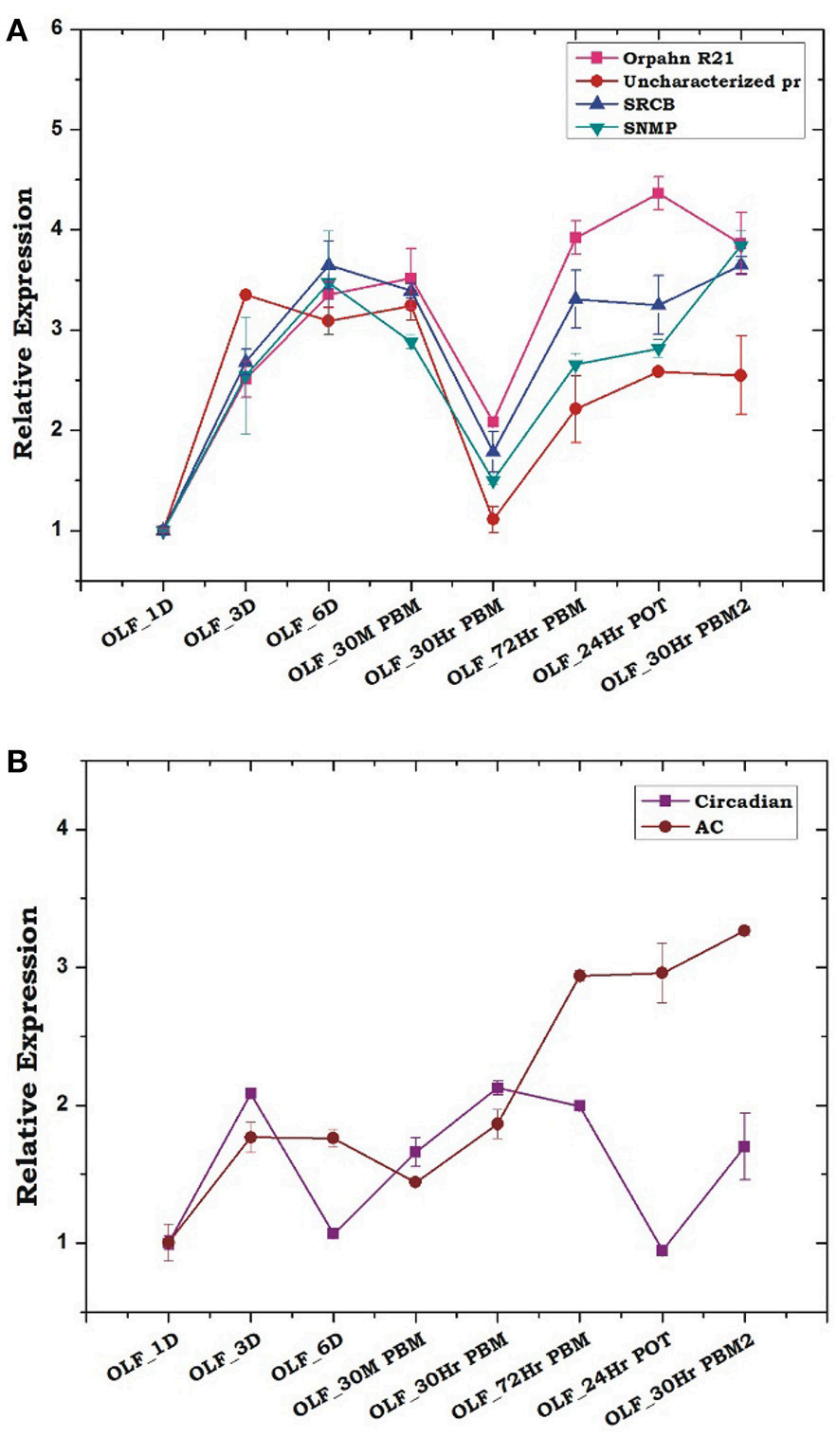
Transcriptional responses of other olfactory genes hypothesized to play a crucial role in host-seeking and blood-feeding behavior. **(A)** Relative expression profiling of other receptor genes according to blood meal time series (described in Figure 5). Orphan R21: Orphan receptor 21; Uncharacterized Pr, Uncharacterized protein; SRCB, Scavenger Receptor class B; SNMP, Sensory neuron membrane protein. **(B)** Transcriptional profiling of other signaling molecule in response to blood meal time series experiment. Circadian, Circadian gene; AC, Adenylyl Cyclase. The significance of suppression of other olfactory genes expression after 30 h of post blood meal are as follows: Orphan R21 ≤ 0.002; Uncharacterized pr ≤ 0.002; SRCB ≤ 0.006; SNMP ≤ 0.007.

Encouragingly, the above data prompted us to test transcriptional profiling of few uncharacterized chemosensory class of olfactory proteins, identified from the transcriptomic data. Transcripts encoding orphan receptor R21, scavenger receptor class B (SRCB), an uncharacterized Protein (XP_001959820) and Sensory neuron membrane protein (SNMP) showed a similar pattern of regulation, suggesting that a combination of all the receptor type represented in the olfactory tissue of *An. culicifacies* mosquito function concurrently in nature's aroma world and changed significantly prior and after the first blood meal as compared to the consecutive second blood meal (Figure 6A). Though, the involvement of G-proteins and related metabotropic signaling mechanism in the olfactory signal transduction of insects remain controversial, however, our observation of a rapid and consistent induction of adenylyl cyclase gene after 30 m PBM (Figure 6B), supported the previous hypothesis that the synthesis of the secondary messenger, cAMP by adenylate cyclase, facilitates odorant mediated signal transduction process which further influence downstream behavioral responses (Takken and Knols, 2010). Surprisingly, finding of <1% of transcripts encoding putative immune proteins suggested that the maintenance of a basal level of sterility is essential for proper olfactory functions (Figure S6).

### Sensory appendages proteins as a unique target to *Anopheles culicifacies*

**Figure 7:**
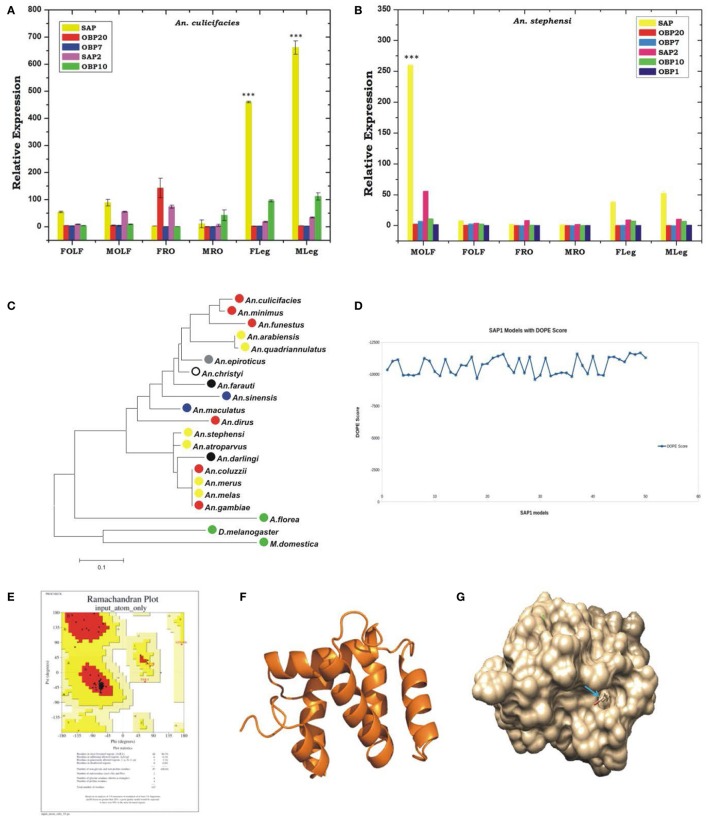
Comparative transcriptional responses of Odorant binding protein genes between two major Indian vectors and structural characterization of one of the potent OBP gene. **(A,B)** Sex and tissue-specific relative expression profiling of OBP genes in *An. culicifacies* **(A)** and *An. stephensi* **(B)**. FOLF, female olfactory tissue (OLF); MOLF, Male OLF; FRO, Female reproductive organ; MRO, Male reproductive organ; FLeg, Female legs; MLeg, Male legs. OBP gene details: SAP, Sensory appendages protein 1; SAP2, Sensory appendages protein 2. **(C)** Phylogenetic analysis of *An. culicifacies* SAP1 (Ac-SAP1) gene. Color-coded circle represents the nature of the mosquitoes host preferences e.g., Red circle, Strongly Anthropophilic; Blue circle, Strongly Zoophilic; Yellow circle, Opportunistic Anthropohilic and/or Zoophilic; Black circle, moderate Anthropophilic; Gray circle, Moderate zoophilic; Blank Circle, Unknown; Green circle, non-blood feeder. **(D)** DOPE score analysis for SAP1. **(E)** Ramachandran Plot of SAP1 protein. **(F)** 3-dimensional protein structure of the Ac-SAP1 protein. **(G)** The binding site of SAP1 protein showed in space fill with nearby residues in stick form. ***p ≤ 0.0001.

To test whether any species-specific olfactory derived genetic factors have any differential regulation likely to influence the behavioral responses, we compared the expression of at least 6 OBPs transcripts between two laboratories reared mosquito species *An. stephensi* and *An. culicifacies*. Though, both are dominant malaria vectors in urban and rural India, respectively, but display a significant difference in their behavioral properties such as feeding, mating, biting preferences etc., (personal observation/ST-S5). In this analysis, we also included two SAP proteins, which showed a high induction than other OBPs in the olfactory system of *An. culicifacies* mosquito at midnight (Figure 4A). Surprisingly, a sex and tissue-specific comparative transcriptional profiling of selected OBPs revealed a dominant expression of SAP1 (*p* ≤ 0.0003)/SAP2 (*p* ≤ 0.0007) in the legs of *An. culicifacies* mosquito (Figures 7A,B). Together these data indicated that *An. culicifacies* may draw an extra advantage of having more sensitive appendages, possibly to favor more active late night foraging behavior, than *An. stephensi*. Though, defining the basis of host preference remains uncertain, because *Anopheline* mosquitoes have opportunistic feeding behavior which is largely influenced by nature of the host availability (Thiemann et al., 2011). A close association of Ac-SAP proteins with the anthropophilic *Anopheline* mosquitoes (Figure 7C, Figure S7A), strongly suggested that sensory appendages proteins may have a crucial role to meet and manage the high host seeking behavioral activities, restricted to *An. culicifacies*.

The above findings further prompted us to carry out a 3D structure modeling analysis of Ac-SAP1 and Ac-SAP2, to predict the best possible conserved binding pockets for specific chemicals. In the absence of any available solved X-ray structure of the reference SAP protein, we applied a template based comparative molecular modeling approach. An initial BLAST analysis identified two best templates in PDB database code for chemosensory protein 2GVS and 1KX8 with identity 47–56% and coverage >80%, favoring their suitability for structure prediction. Out of the 50 modeled 3D structures for each protein, DOPE score analysis resulted in the selection of model-49 and model-27 with score −11689.73, and −10989.75 for SAP1 and SAP2, respectively (Figure 7D, Figure S7B).

We validated the best-selected model using Procheck server for Ramachandran plot, showing a more than 95% allowable region, with no residue falling in the disallowed region of the plot (Figure 7E, Figure S7C). Based on the consensus, a best-fit ligand binding site prediction analysis within the selected models was scored by COACH server, which engages at least five different algorithms TM-SITE, S-SITE, COFACTOR, FIND-SITE, and ConCavity. Binding pocket for SAP1 and SAP2 identified eight consensus residues namely D36, E39, L40, K49, C52, Q59, Y91, and Y95 along with BDD (12-bromo-1-dodecanol) as a predicted ligand. Minimization of the steric clashes from the complex structures was done using Chimera software (Figure 7F, Figure S7D). Furthermore, selection of amino acid residues within 3 Å region of ligand molecule are I43 and Y95 of which Y95 is involved in H-bonding with BDD ligand. Similarly, in case of SAP2 protein, residue selection resulted in the identification of I43, D51, Q59, T63, Y95 residues of which D51 form H-bond with BDD ligand (Figure 7G, Figure S7E).

In our analysis, we observed the presence of at least two conserved cysteines (CYS52 and CYS55) residues in the loop region of SAP1 and SAP2 proteins, which may likely have involved in di-sulfide bond formation and stabilization of protein structure. Our analysis also showed that binding pocket forms a tunnel-like structure which is preferred by long aliphatic molecules. Presence of negatively charged aspartic and glutamic acid at both ends showed the preference for charged residue near the vicinity of ligand molecule. Moreover, the presence of conserved negatively charged aspartic acid and polar tyrosine (TYR91 in SAP1 and TYR95 in SAP2) at one end of binding pocket suggested their role in ligand binding.

## Discussion

It is well known that a circadian dependent modulation of olfactory responses significantly influences the complex behavioral responses in both sexes of *Anopheline* mosquito species (Rund et al., 2013). However, the evolution of the more specialized olfactory system of adult female mosquitoes favored their unique adaptation to blood feeding behavior. The female olfactory system comprises of three olfactory appendages i.e., (a) the antennae, (b) the maxillary palps and (c) female specific proboscis, which may encode a variable number of factors responsible for maintaining female specific daily olfaction rhythms such as host seeking, blood feeding, and oviposition behavior. Since, peripheral antennal appendages harbor more diverse OBPs, Ors, and other factors, it acts as the principle chemosensory organ that respond to wide range of volatile odors. Therefore, major electrophysiological and molecular studies have been focussed on this olfactory component. A few recent studies examining global profile change in response to daily rhythms and blood feeding highlighted the important role of the antennal transcripts/proteins in the modulation of distinct behavioral responses of *Anopheline* mosquitoes (Rinker et al., 2013; Rund et al., 2013; Chen et al., 2017). While the maxillary palps encode unique receptor proteins such as Or8, Or28, and Orco, which respond to carbon dioxide to enable mosquitoes for a successful navigation toward vertebrate hosts (Pitts et al., 2011). In the close vicinity of the targeted hosts, female mosquito's proboscis encoded factors rapidly engaged to complete the blood meal uptake process in less than 2 min. The molecular basis that how olfactory appendages encoded factors interlinked with each other to drive highly sex-specific pre-and post-blood meal behavioral events are not well understood yet.

We have recently demonstrated that adult female mosquitoes are evolved with the unique ability of salivary gland gene expression switching to manage meal specific “prior and post” blood meal responses (Sharma et al., 2015b). Here, we further extended this idea to decode and trace the possible molecular link that how the olfactory factors of adult female *An. culicifacies* mosquitoes drive sex-specific host-seeking, blood-feeding and oviposition behavior. To establish the plausible mechanism of the olfactory system, we developed a working hypothesis (Figure 2) and compared the transcriptional response of the olfactory derived transcripts, modulating in responses to changes in the feeding status. Surprisingly, an observation of a limited change in the global response of the olfactory system of *An. culicifacies* mosquito partly corroborates with the similar changes in the limited pool of antennal chemosensory genes in *An. gambiae* (Rinker et al., 2013). Taking in account of the nature of tissues i.e., the selected peripheral sensory appendages investigated in previous studies, we hypothesize that blood-feeding may not directly cause a major shift in transcript abundance, but may alter the functional nature/regulation of the unique transcripts controlling key biological processes. To unravel the molecular nature and function of the olfactory factors, we annotated, cataloged and selectively profiled the expression of OBPs, Ors and other members of chemosensory genes.

An initial comparison of the annotated transcripts revealed that first blood meal not only delimits the transcripts numbers but also enriches the expression of many unique transcripts having similar functions. Once reached to its saturation level, the expression of selected olfactory transcripts did not alter significantly, when offered an un-interrupted sugar meal to the aging mosquitoes (Figures S8A,B). Together, these data suggested that an abundant expression of olfactory receptors in naïve mosquitoes may be essential to encounter and manage different conflicting behavioral demands when changing from naïve sugar fed to blood fed status. Furthermore, a zeitgeber time scale experiments suggested that midnight hyper activities of OBPs, especially sensory appendages proteins (SAP-1 and SAP-2), are able to drive female specific host-seeking behavioral activities of naive adult female *An. culicifacies* mosquitoes, supporting the previous finding in other *Anopheline* mosquito species (Biessmann et al., 2005; Iovinella et al., 2013).

Our observation of a transient change in the expression of selected OBP transcripts, in response to first blood meal further raises a question that how mosquitoes manage blood feeding associated complex behavioral responses such as egg maturation, oviposition etc. After a successful blood meal, the gut physiology of the naive adult female mosquito undergoes a complex modulation to digest the blood meal and maturation of the eggs. Once the blood meal digestion completed, the mosquitoes may re-switch their olfactory responses for oviposition site finding behavior (Wong et al., 2011; Phasomkusolsil et al., 2013; Rinker et al., 2013; Lindh et al., 2015). Current literature suggested that a combinatorial coding mechanism of the olfactory receptors enables insects to recognize thousands of diverse chemical cues for selective neuro-actions to meet specific behavioral demands (Carey and Carlson, 2011; Martin et al., 2011; Andersson et al., 2015). Though previous studies suggested that first blood meal causes the alteration of OBP/Ors mediated odor sensitivity (Rinker et al., 2013), how olfactory receptors superintend and co-ordinate between innate and primed/adaptive odor responses remains largely unknown (Lutz et al., 2017). We hypothesize that a harmonious action of OBPs and Ors, which are involved in downstream odorant signal transduction cascade, may have significant influence on behavioral switching events.

To test this hypothesis, we profiled the expression of selected Ors transcripts in response to two consecutive blood meal follow up experiment, which included at least one gonotrophic cycle completion. Supporting the previous reports, we also observed that a first blood meal initiated a gradual suppression of all the olfactory receptor transcripts within 30 min of blood feeding, which was further ceased to the basal level at 30 h post blood meal. However, surprisingly, we observed a two-fold up regulation of all the receptor transcripts in response to second blood meal, when compared the expression after 30 h of first blood meal. Together, these data strongly suggested that first blood meal exposure to odorant receptors may have priming effect over host-seeking behavioral activities, enabling mosquito for rapid blood meal uptake for consecutive gonotrophic cycles.

Two most potent Indian malarial vector species *An. culicifacies and An. stephensi* have been reported to show predominantly zoophilic and anthropophilic behavior, respectively (Joshi et al., 1988; World Health Organization, 2007; Sharma and Dev, 2015), but the molecular basis of such biological variation is yet to unravel. Emerging evidence suggested that a significant genetic difference exists among various *Anopheline* mosquito species, including *An. stephensi* and *An. culicifacies* (Dash et al., 2007; Sharma et al., 2015a). Under laboratory investigation, we frequently observed that biological rhythm may have a significant influence on the biting and blood feeding behavior of *An. culicifacies*. Previously, several odorant binding proteins such as OBP20/OBP1/OBP7, SAP have been identified and characterized as a key molecular target in many *Anopheline* mosquitoes involved in host-seeking behavior (Biessmann et al., 2010; Sengul and Tu, 2010; Ziemba et al., 2013), but remains poorly understood in Indian vectors.

Therefore, to test whether species-specific olfactory derived genetic factors have any differential regulation, we compared the tissue expression of selected OBP transcripts between two laboratories reared mosquito species i.e., *An. stephensi* and *An. culicifacies*. Surprisingly, a higher expression of SAP-1 and SAP-2 in the legs of mosquito *An. culicifacies* indicated that this mosquito species may have drawn an extra advantage of having more sensitive appendages, possibly to favor more active late night foraging behavior than *An. stephensi*. A 3D molecular modeling analysis not only predicted the presence of at least two conserved cysteines (CYS52 and CYS55) residues in the loop region of SAP1 and SAP2 proteins but also suggested that binding pocket may form a tunnel-like structure, preferred by long aliphatic molecules. While the presence of conserved negatively charged aspartic acid and polar tyrosine at one end of binding pocket suggested their role in ligand binding. Though previously SAP has also been identified from other *Anopheline* mosquito species but their role in host-seeking and blood feeding behavior remains poorly understood (Biessmann et al., 2005; Iovinella et al., 2013). Encouraged by the above observation, we selected SAP as a unique target that may be crucial to design an effective disorientation strategy against *An. culicifacies* mosquito, an important malaria vector in rural India.

## Conclusion

**Figure 8:**
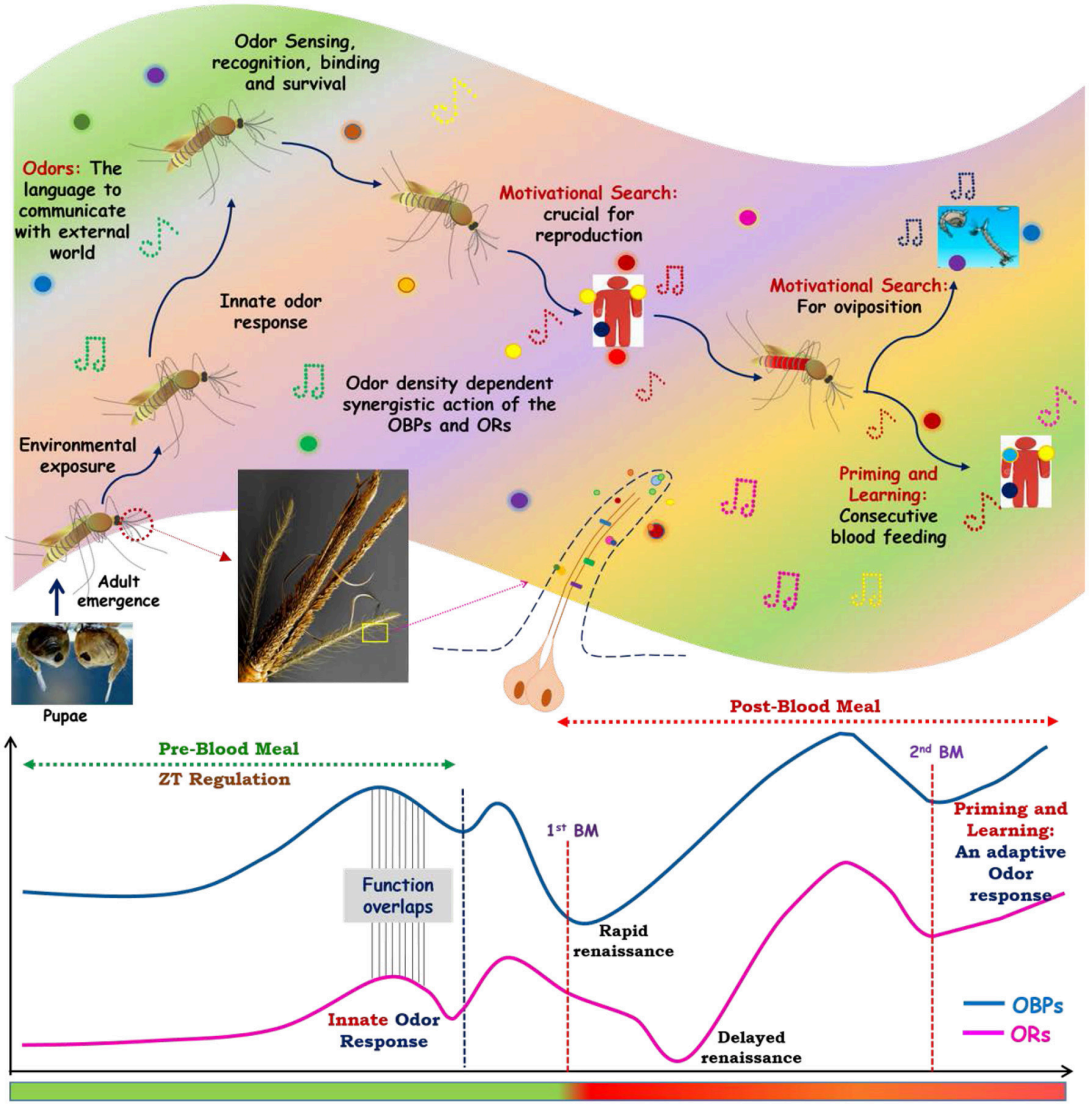
How smart actions of olfactory system manages blood feeding associated odor response: an evolutionary speciality of adult female mosquitoes. After emergence from pupae adult mosquitoes are exposed to the overwhelmed odor world, where odorants chemicals act as a language of communication with the external world. The sophisticated innate olfactory system of mosquitoes enables them to recognize and differentiate this wide variety of odorants which are crucial for their every life cycle stages. Inner physiological motivation, as well as the age and exposure of mosquitoes toward the external world, promote them for host seeking and blood feeding event. After taking blood meal mosquitoes initiate next level of physiological cum behavioral events i.e., oviposition. Apart from that, first exposure to vertebrates facilitates learning and second blood feeding events. These whole odors mediated response is tactfully managed by the synergistic actions of Odorant binding proteins (OBPs) and olfactory receptors (Ors). The overlapping circadian rhythm dependent functions of OBPs and Ors govern the pre-blood meal events of host fetching events. As soon as the mosquitoes take blood meal the functions of OBPs and Ors ceased for some period, but the recovery of OBPs actions occurs early as compared to Ors to perform the next level of behaviors. Mosquitoes, then take advantage/adapted from priming and learning of the first blood meal exposure for the more rapid consecutive blood feeding.

Decoding the genetic relationship of sense of smell is central to design new molecular tools to disrupt mosquito-human interaction. We demonstrated that a synergistic and harmonious action of olfactory encoded unique factors govern the successful “prior and post” blood feeding associated behavioral complexities. A comprehensive RNA-Seq and extensive transcriptional profiling data, further strengthen the hypothesis that a quick recovery of the actions of odorant binding proteins immediately after blood feeding, and delayed re-activation of olfactory receptor proteins after blood meal digestion completion are unique to manage diverse behavioral responses. However, an extended blood meal follows up experimental data analysis further hypothesize that first blood meal exposure is enough for prime learning, satisfying the motivational search of mosquitoes for the completion of their gonotrophic cycles. Thus, it is plausible to propose that apart from the innate odor responses, adult female mosquitoes might take an advantage of prior odor (vertebrate) exposure, which leads an exclusive evolutionary specialty, allowing them to learn, experience and adapt as a fast blood feeder in nature (Figure 8).

In summary, we decoded and established a possible functional correlation that how coherent and smart actions of olfactory encoded factors enabled adult female mosquitoes to meet and manage the blood feeding associated complex behavioral activities (Figure 8). Furthermore, targeting species-specific unique genes such as sensory appendages proteins may be crucial to design disorientation strategy against mosquito *An. culicifacies*, an important malarial vector in rural India.

## Data deposition

The sequencing data were deposited to National Center for Biotechnology Information (NCBI) Sequence Reads Archive (SRA) system (BioProject accessions: PRJNA414162; BioSample accessions: SAMN07981002, SAMN07972755, and SAMN07775994).

## Author contributions

Conceived and designed the experiments: TD, RD; Performed the experiments: TD, TT, SV, DS, VS, PS, CC, SK, ST, JR; Analyzed the data: TD, RD; YH; Contributed reagents, materials, analysis tools: YH, RD, KP; Wrote the paper: TD, RD, YH, KP.

### Conflict of interest statement

The authors declare that the research was conducted in the absence of any commercial or financial relationships that could be construed as a potential conflict of interest.
