# Supplementary material for: A Synergistic Transcriptional Regulation of Olfactory Genes Drives Blood-Feeding Associated Complex Behavioral Responses in the Mosquito Anopheles culicifacies
Source: Front Physiol. 2018 May 23;9:577. doi: 10.3389/fphys.2018.00577 (PMC5974117; doi:10.3389/fphys.2018.00577)
Supplement: Supplementary file 1 [file Data_Sheet_1.docx]

**Supporting Information:**

**A synergistic transcriptional regulation of olfactory genes derives complex behavioural responses in the mosquito *Anopheles culicifacies***

Tanwee Das De ^1, 2^, Tina Thomas^1^, Sonia Verma, Deepak Singla, Vartika Srivastava^1^, Punita Sharma^1^, Charu Rawal^1^, Seena Kumari^1^, Sanjay Tavetiya^1^, Jyoti Rani^1^, Yasha Hasija ^2^, Kailash C Pandey^1,3^ and Rajnikant Dixit ^1*^.


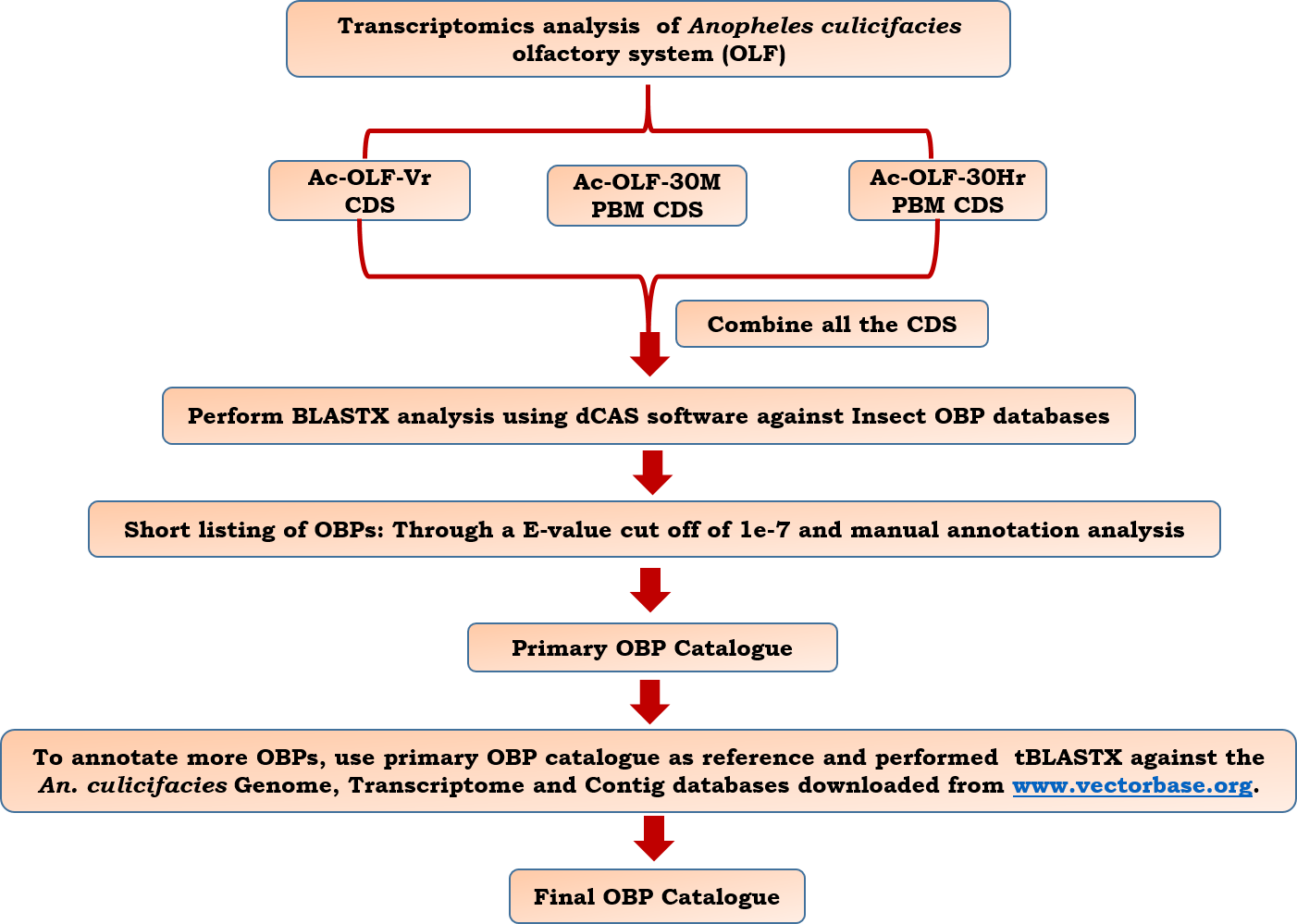


**Fig. 1: Bioinformatic workflow followed to identify the odorant binding protein (OBPs) genes of *Anopheles culicifacies* mosquito.** Putative OBP transcripts were identified from olfactory tissue transcriptome database by performing BLASTX analysis against the insect OBP database using dCAS software package with an E-value cutoff 1e^-7^. To identify and annotate more putative OBP genes from genome of *An. culicifacies*, we performed tBLASTX analysis of all shortlisted OBPs as a query against genome database, downloaded from [www.vectorbase.org](http://www.vectorbase.org/). Removal of all kinds of repetition was done and prepare the final OBP catalogue.


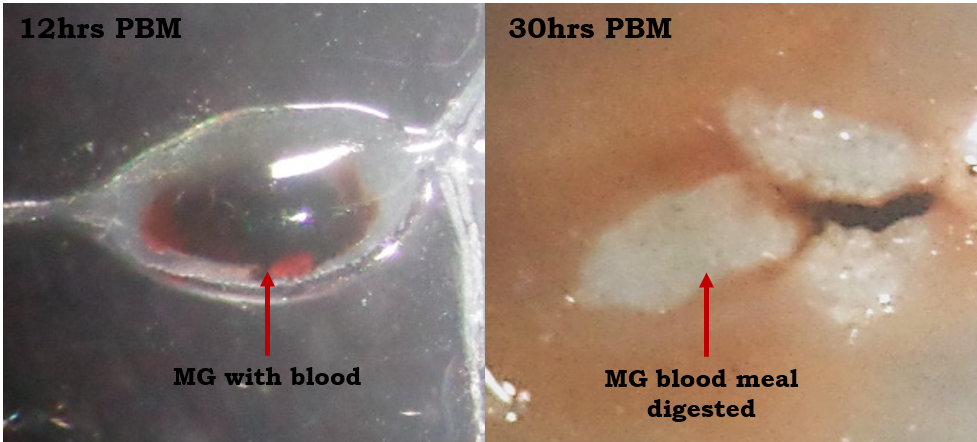


**Fig. S2:** Time dependent depiction of blood fed midgut (MG) showing blood meal digestion. MG digested from 12hrs post blood meal (PBM) showed undigested blood still present in the MG but after 30hrs of PBM blood meal digestion completed and thus no blood remnants is observable.


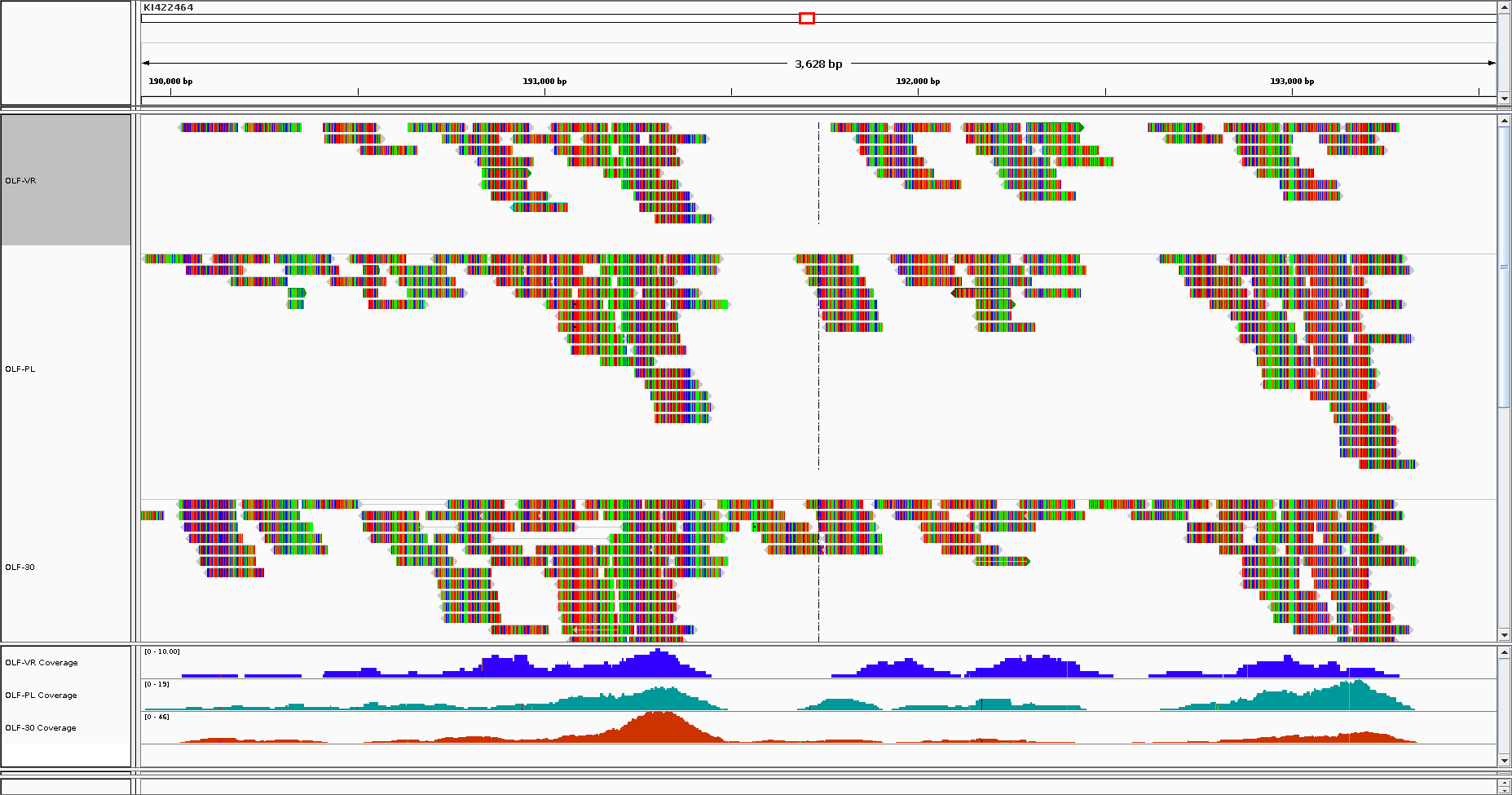


**Figure S3:** Self-explanatory pictorial presentation of genome guided reference mapping limitations of olfactory database of *Anopheles culicifacies*.

**Fig. S4a: Phylogenetic analysis of Classic-OBPs. Classic-OBPs of *An. culicifacies*** showed conserved sequence relationship with *An. gambiae* and other mosquito and insect species. Different color code that indicating a particular mosquito and insect species: Blue – *An. gambiae*; Red – *An. culicifacies*, Green – *Culex quinquefasciatus*. *Drosophila melanogaster* (DRX) and *Aedes aegypti* (AAX) are not marked with any color code.

**Fig. S4b: Phylogenetic analysis of Plus-C OBP superfamily.** *An. culicifacies* Plus-C class of OBPs are more closely linked to mosquito specific OBPs and showed distant relationship to other non-mosquito species for example *Drosophila.* Different color code that indicating a particular mosquito and insect species: Blue – *An. gambiae*; Red – *An. culicifacies*, Green – *Culex quinquefasciatus*; Sky blue – *D. melanogaster. Aedes aegypti* (AAX) is not marked with any color code.

**Fig. S4c: Phylogenetic analysis of Atypical OBP superfamily.** *An. culicifacies* Atypical OBP class are more closely linked to different mosquito species and showed distant relationship to other non-mosquito species for example *Drosophila.* Different color code that indicating a particular mosquito and insect species: Blue – *An. gambiae*; Red – *An. culicifacies*, Pink – *Culex quinquefasciatus*; Sky blue – *D. melanogaster;* Green - *Aedes aegypti*.


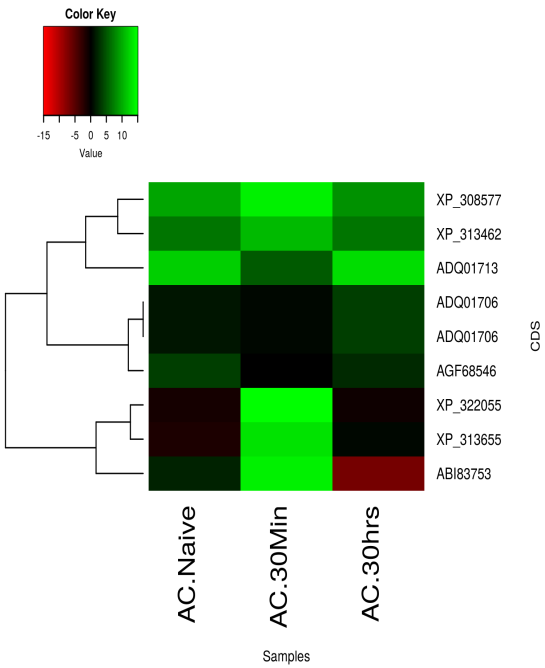


**Figure S5: Transcriptional Response of *An. culicifacies* OBPs. (**a) Heat map showing differential expression pattern of eight common OBP genes in naïve and blood fed olfactory tissue of *An. culicifacies*.


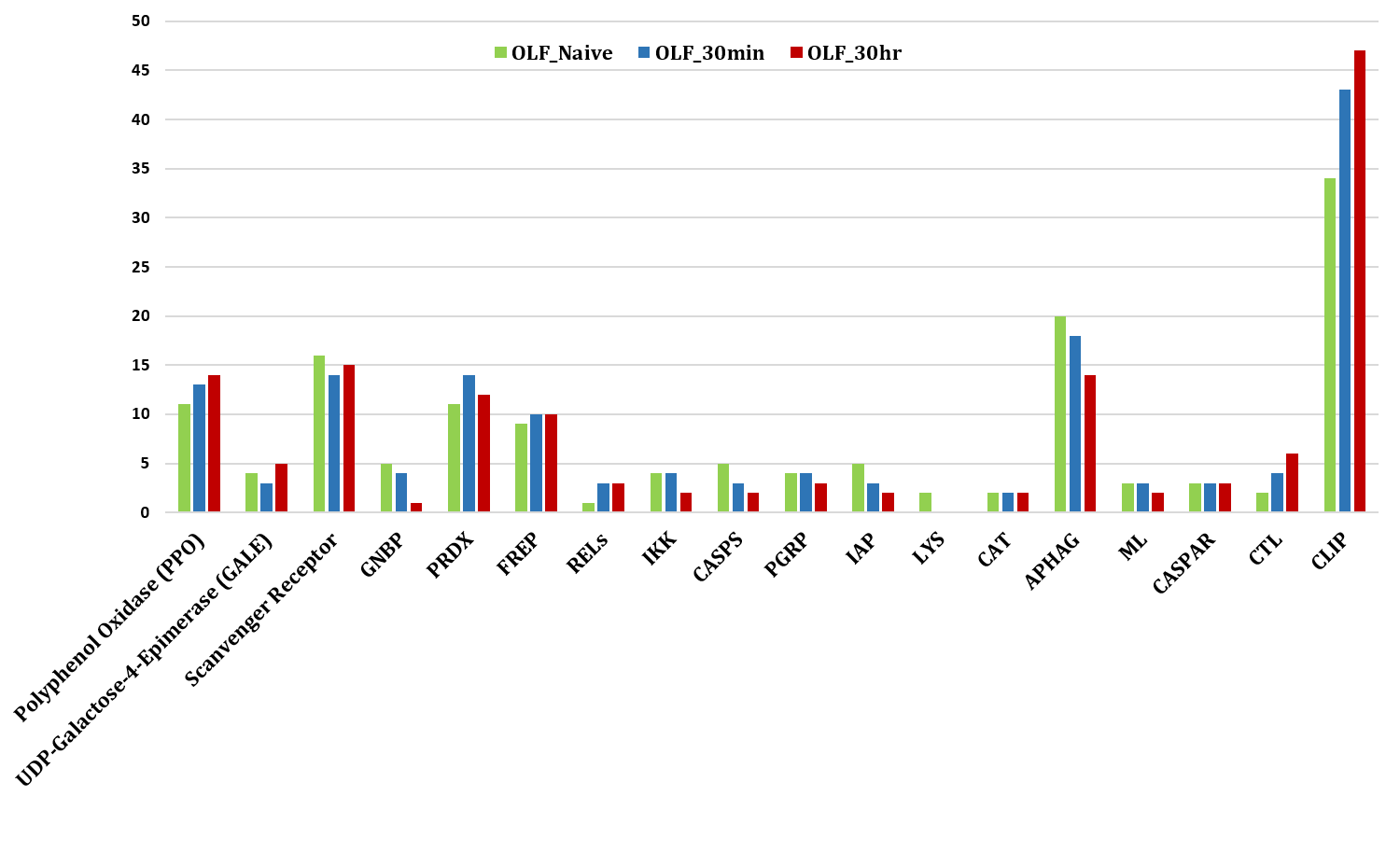


**Figure S6: Differential expression pattern of olfactory specific immunome.** The immune genes expressed in the olfactory system of *An. culicifacies* are retrieved through BLASTX analysis against the insect ImmunoDB database and categorized into eighteen different family members and their differential expression pattern was determined by the number of sequences appeared in each RNASeq data of naïve and blood fed mosquitoes.


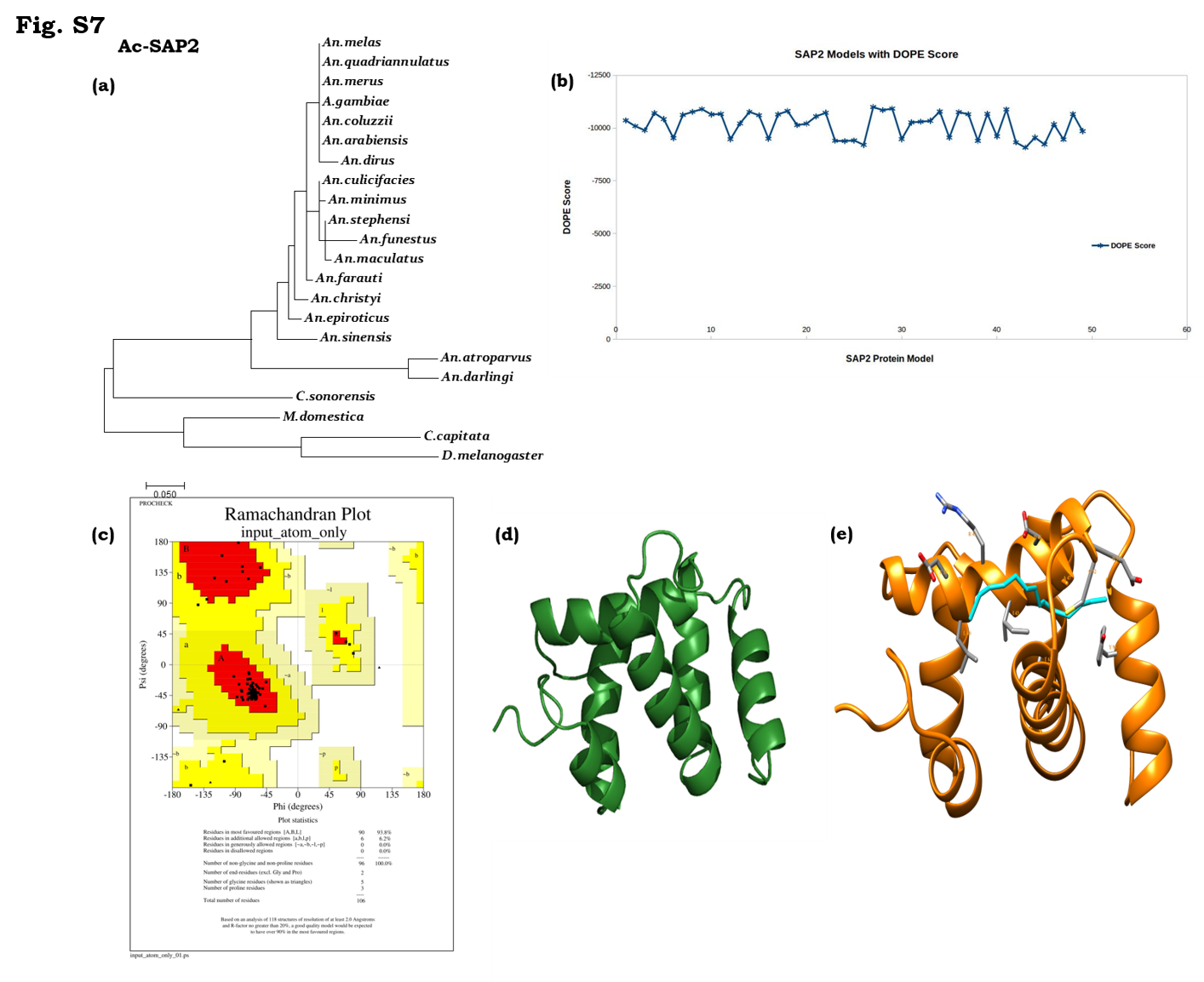


**Figure S7:** **Phylogenetic and structural analysis of SAP2**. (a)Phylogenetic analysis of *An. culicifcaies* SAP2 gene (Ac-SAP2). (b) DOPE score analysis for SAP2. (c) Ramachandran Plot of SAP2 protein. (d) 3-dimentional protein structure of Ac-SAP2 protein. (e) Binding site of SAP2 protein showed in space fill with nearby residues in stick form.


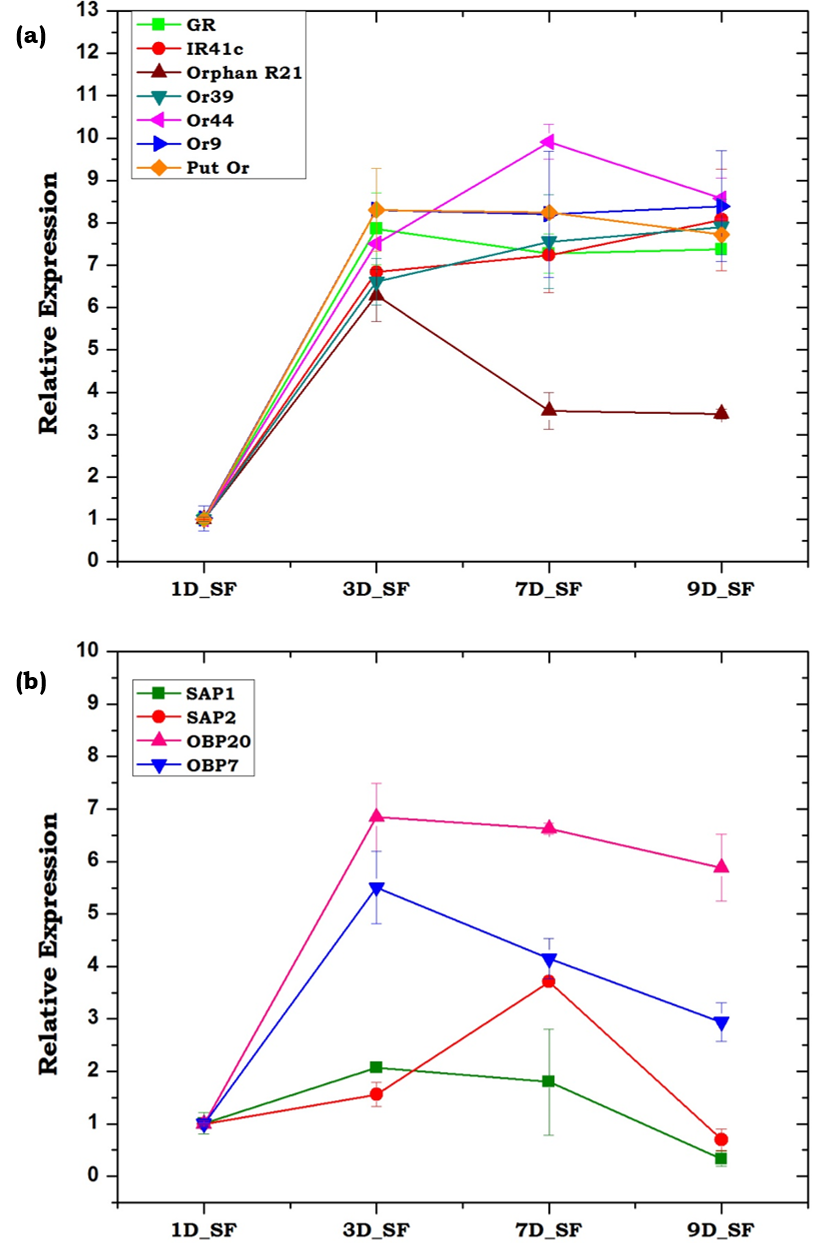


**Figure S8:** An un-interrupted sugar meal does not alter the Ors (a) and OBPs (b) expression in aging mosquitoes. Post emergence the adult female mosquitoes were kept on sugar meal and olfactory system were collected till nine day. A relative expression was profiled by real-time PCR.

**Supplementary Table 1: Annotation kinetics of the RNA-Seq data**

| **Molecular Features** | **Ac-OLF-Naive** | **Ac-OLF-30M PBM** | **Ac-OLF-30Hr PBM** |
| --- | --- | --- | --- |
| **Total Transcripts** | 8133 | 8907 | 8396 |
| **Total BLASTx hits (NR)** | 7245 (~89%) | 7553 (~84.79%) | 6829 (~81.33%) |
| **Transcripts with GO Match** | | | |
| **Molecular Function** | 3946 | 4137 | 3763 |
| **Biological process** | 3804 | 3985 | 3566 |
| **Cellular component** | 2097 | 2201 | 1999 |
| **Transcript with KEGG match** | 2645 (~32.52%) | 2975 (~33.40%) | 2740 (~32.63%) |

**Supplementary Table 2: Percentage of Differentially Expressed Transcripts**

| **Sample** | **No. of Transcripts** | **Transcripts showing Differential gene Expression (DGE)** | **Upregulated Transcripts** | **Downregulated Transcripts** | **Percentage of Transcripts showing DGE** |
| --- | --- | --- | --- | --- | --- |
| Ac_OLF_naive vs Ac_OLF_30min | (8133 +  8907) = 17040 | Total - 3749  Significant – 2540  Not significant - 1209 | 1042 (6%) | 1498 (8.7%) | 14.9% CDS show differential expression |
| Ac_OLF_Naive vs Ac_OLF_30hr | (8133 +  8396) = 16529 | Total -3377  Significant – 2128  Not significant - 1249 | 396 (2%) | 1732 (10%) | 12.87 % show differential expression |

**Supplementary Table S3:**

| **Serial No.** | **Transcript ID** | **Origin of the Transcript** | **Name** | ***An.culicifacies* Database Match** | **E-Value** | **Identity** | **Sub-Family** | **Ortholog in *An.gambiae*** | **FPKM** |
| --- | --- | --- | --- | --- | --- | --- | --- | --- | --- |
|  | Transcript_1018 | 30hr PBM | OBP20 | ACUA000236 | 7E-63 | 95.70% | Classic | AGAP005208 | 3173.27822520792 |
|  | Transcript_1216 | 30hr PBM | GOBP72 | ACUA019958 * | 6.00E-57 | 99% | Classic | AGAP012714 | 1254.82586 |
|  | Transcript_127 | 30hr PBM | OBP7 | ACUA012847 | 2.00E-85 | 98% | Classic | AGAP001556 | 4144.93009 |
|  | Transcript_1450 | 30hr PBM | OBP26 | ACUA003587 * | 3.00E-80 | 98.50% | Classic | AGAP012321 | 2051.81759 |
|  | Transcript_4885 | 30hr PBM | OBP63 | ACUA020502 * | 6.00E-70 | 99.30% | Classic | AGAP012322 | 187.690564 |
|  | Transcript_6613 | 30hr PBM | OBP3 | ACUA024106 | 5.00E-07 | 92.30% | Classic | AGAP001409 | 21144.6696 |
|  | Transcript_91 | 30hr PBM | GOBP71 | ACUA003567 | 9.00E-169 | 95.50% | Classic | AGAP012331 | 4939.85295 |
|  | Transcript_109 | Naive | OBP5 | ACUA003763 | 2.00E-48 | 97.60% | Classic | AGAP009629 | 301.156325 |
|  | Transcript_3113 | Naive | OBP28 | ACUA001691 | 1.00E-75 | 96.20% | Classic | AGAP012325 | 63.5528511 |
|  | Transcript_1290 | Naive | OBP9 | ACUA003296 | 6.00E-89 | 100% | Classic | AGAP000278 | 389.876797 |
|  | Transcript_138 | Naive | OBP6 | ACUA004501 * | 3.00E-12 | 41.20% | Classic | AGAP003530 | 238.355131 |
|  | Transcript_270 | Naive | OBP2 | ACUA004189 | 8.00E-90 | 97.30% | Classic | AGAP003306 | 211.717742 |
|  | Transcript_218 | 30min_PBM | OBP10 | ACUA017068 | 1.00E-71 | 99.20% | Classic | AGAP001189 | 40.2405414 |
|  | Transcript_48 | 30min_PBM | OBP25 | ACUA014063 | 3.00E-65 | 99.20% | Classic | AGAP012320 | 92.3071105 |
|  | Transcript_62 | 30min_PBM | OBP1 | ACUA014299 | 2.00E-87 | 99.30% | Classic | AGAP003309 | 608.296339 |
|  | Transcript_59 | 30min_PBM | OBP54 | ACUA001499 * | 2.00E-56 | 97.10% | Pluc-C | AGAP006080 | 41.4381766 |
|  | Transcript_3357 | 30min_PBM | GOBP69 | ACUA024897 | 3.00E-94 | 93% | Pluc-C | AGAP013182 | 323.549283 |
|  | Transcript_207 | 30min_PBM | OBP47 | ACUA011874 | 2.00E-115 | 96.90% | Pluc-C | AGAP007287 | 116.931 |
|  | Transcript_7419 | 30min_PBM | OBP58 | ACUA019212 * | 1.00E-109 | 91.60% | Pluc-C | AGAP006074 | 28.093679 |
|  | Transcript_5393 | 30min_PBM | OBP46 | ACUA020810 | 8.00E-123 | 95.60% | Pluc-C | AGAP007289 | 23.4694097 |
|  | Transcript_6620 | 30min_PBM | OBP56 | ACUA025325 | 1.00E-143 | 97.40% | Pluc-C | AGAP011367 | 20.576198 |
|  | Transcript_611 | Naive | OBP57 | ACUA028407 | 1.00E-114 | 97.60% | Pluc-C | AGAP011368 | 1983.04862 |
|  | Transcript_4488 | Naive | OBP51 | ACUA023815 * | 7.00E-23 | 87.50% | Pluc-C | AGAP006077 | 65432.854 |
|  | Transcript_22 | Naive | OBP48 | ACUA025211 | 3.00E-59 | 87.30% | Pluc-C | AGAP007286 | 1728.56394 |
|  | Transcript_853 | 30hr PBM | OBP43 | ACUA001907 | 0.00E+00 | 96.30% | Two Domain | AGAP009402 | 90.5977525 |
|  | Transcript_2542 | 30hr PBM | D7 | ACUA025281 * | 0 | 98.70% | D7 Protein Family | AGAP006278 | 462.002781 |
|  | Transcript_2682 | 30hr PBM | CSP | ACUA008309 * | 1.00E-66 | 100% | Chemosensory Protein | AGAP001303 | 215.86399 |
|  | Transcript_5645 | 30hr PBM | LD7 | ACUA002223 | 3.00E-77 | 98.40% | D7 Protein Family | AGAP007286 | 34824.3385 |
|  | Transcript_64 | 30hr PBM | SD7 | ACUA001093 | 1.00E-33 | 98.40% | D7 Protein Family | AGAP008281 | 1348.33451 |
|  | Transcript_1808 | Naive | CSP | ACUA010232 * | 6.00E-51 | 96.70% | Chemosensory Protein | AGAP008055 | 519.825389 |
|  | Transcript_447 | Naive | CSP3 | ACUA004458 | 7.00E-71 | 96.70% | Chemosensory Protein | AGAP008059 | 195.413717 |
|  | Transcript_702 | Naive | CSP | ACUA017151 | 1.00E-29 | 90.90% | Chemosensory Protein | AGAP008054 | 203.377554 |
|  | Transcript_12 | Naive | Sensory Appendage Protein | ACUA018714 | 2.00E-40 | 100% | Chemosensory Protein | AGAP008051 | 24346.8834 |
|  | Transcript_5005 | Naive | LD7 | ACUA018525 | 9.00E-100 | 97.70% | D7 Protein Family | AGAP008279 | 53.6723049 |
|  | Transcript_631 | Naive | LD7 | ACUA008578 | 1.00E-73 | 89.70% | D7 Protein Family | AGAP028120 | 27.625451 |
|  | Transcript_1446 | 30min_PBM | CSP | ACUA013563 * | 5.00E-80 | 99.40% | Chemosensory Protein | AGAP008058 | 149.776542 |

**Genome Annotated OBP**

| **Serial No.** | ***An.culicifacies* Transcript ID** | **Name** | **Signal Peptide** | **OBP Subfamily** | **Ortholog in**  ***An.gambiae*** |
| --- | --- | --- | --- | --- | --- |
|  | ACUA027288 | OBP11 | Yes | Classic | AGAP002025 |
|  | ACUA007455 * | OBP64 | Yes | Classic | AGAP012324 |
|  | ACUA019075 | OBP18 | Yes | Classic | AGAP012319 |
|  | ACUA007086 * | OBP62 | Yes | Classic | AGAP002556 |
|  | ACUA016883 | OBP13 | Yes | Classic | AGAP002905 |
|  | ACUA005593 | OBP23 | Yes | Classic | AGAP012318 |
|  | ACUA011194 | OBP21 | Yes | Classic | AGAP008398 |
|  | ACUA019079 | OBP12 | Yes | Classic | AGAP002188 |
|  | ACUA022547 | OBP8 | No | Classic | AGAP000279 |
|  | ACUA009276 | OBP27 | Yes | Classic | AGAP012323 |
|  | ACUA018051 | OBP22 | No | Classic | AGAP010409 |
|  | ACUA023151 * | OBP60 | No | Plus-C | AGAP007281 |
|  | ACUA006032 | GOBP70 | Yes | Plus-C | AGAP006368 |
|  | ACUA005261 | GOBP68 | Yes | Plus-C | AGAP012658 |
|  | ACUA013374 | GOBP67 | Yes | Plus-C | AGAP007282 |
|  | ACUA003780 * | OBP44 | Yes | Two Domain | AGAP010648 |
|  | ACUA011054 * | OBP31 | Yes | Two Domain | AGAP010649 |
|  | ACUA011785 | OBP45 | Yes | Two Domain | AGAP010650 |
|  | ACUA011802 * | OBP35 | Yes | Two Domain | AGAP000642 |
|  | ACUA004895 | OBP42 | Yes | Two Domain | AGAP009065 |
|  | ACUA010086 * | OBP36 | Yes | Two Domain | AGAP000643 |
|  | ACUA020837 | OBP32 | Yes | Two Domain | AGAP000640 |
|  | ACUA028423 * | OBP40 | Yes | Two Domain | AGAP002191 |
|  | ACUA008136 * | OBP38 | No | Two Domain | AGAP000580 |
|  | ACUA017746 * | OBP39 | Yes | Two Domain | AGAP002190 |
|  | ACUA003948 | OBP41 | Yes | Two Domain | AGAP005182 |
|  | ACUA016063 * | OBP37 | No | Two Domain | AGAP000641 |

***Newly annotated OBPs.**

**Supplemental Table S4: Catalogue of Olfactory Receptor:**

| **Sl No.** | **Gene Accession No.** | **Receptor Type** | ***An. culicifacies* ID** | ***An. gambiae* Identity** | **CDS Origin** | **FPKM** |
| --- | --- | --- | --- | --- | --- | --- |
| 1. | XP_310061 | Or29 | ACUA025107 | AGAP009111 | Naïve_Transcript_6279 | 26.602643 |
| 2. | XP_315068 | OR35 | ACUA009020 | AGAP004971 | Naive_Transcript 7874 | 11.9370834 |
| 3. | XP_315072 | OR31 | ACUA014252 | AGAP004974 | Naive_Transcript_4545 | 27.0352063 |
| 4. | XP_309205 | OR36 | ACUA003785 | AGAP001012 | Naive_Transcript_3706 | 14.6902145 |
| 5. | XP_311894 | OR62 | ACUA008089 | AGAP011978 | Naïve_Transcript_4436 | 17.7898333 |
| 6. | XP_313640 | OR26 | ACUA020087 | AGAP004357 | Naive_Transcript_7012 | 19.519759 |
| 7. | XP_315773 | OR33 | ACUA020367 | AGAP005760 | Naive_Transcript_6630 | 19.2215628 |
| 8. | XP_317124 | OR9 | ACUA008607 | AGAP008333 | Naive_Transcript_6764 | 129.219612 |
| 9. | XP_321150 | Gustatory Receptor | ACUA025789 | AGAP001915 | Naive_Transcript_6229 | 39.039518 |
| 10. | EFR27310 | IR21a | ACUA000154 | AGAP008511 | Naive_Transcript_5794 | 8.28742772 |
| 11. | XP_311997 | IR41a | ACUA014728 | AGAP002904 | Naive_Transcript_7850 | 23.907604 |
| 12. | XP_003436511 | IR41c | ACUA016051 | AGAP012951 | Naive_Transcript_2012 | 1286.70859 |
| 13. | EFR22053 | Ionotropic Receptor NMDAR3 | ACUA007915 | AGAP005527 | Naïve_Transcript_5355 | 10.5566044 |
| 14. | XP_312117 | GLURIIc Ionotropic Receptor | ACUA017915 | AGAP002797 | Naive_Transcript_6513 | 32.6012782 |
| 15. | XP_312026 | Putative GPCR class Orphan Receptor | ACUA015630 | AGAP002886 | Naive_Transcript_6435 | 17.9142377 |
| 16. | XP_311816 | OR45 | ACUA019417 | AGAP003053 | Naive_Transcript_2629 | 21.5803151 |
| 17. | XP_003436373 | Odorant Receptor | ACUA019417 | AGAP013396 | Naive_Transcript_7336 | 13.7599877 |
| 18. | XP_318786 | OR72 | ACUA020212 | AGAP009718 | Naive_Transcript_4446 | 7.45172072 |
| 19. | AGS08024 | OR41 | ACUA014638 | AGAP000226 | Naive_Transcript_5372 | 39.3789123 |
| 20. | XP_310066 | OR16 | ACUA017005 | AGAP009394 | 30min PBM_Transcript_4012 | 22.9711849 |
| 21. | XP_312289 | OR 39 | ACUA018263 | AGAP002639 | 30min PBM_Transcript_1117 | 151.09935 |
| 22. | XP_315048 | OR32 | ACUA026067 | AGAP004951 | 30min PBM_Transcript_8291 | 46.7776857 |
| 23. | XP_320543 | Or63 | ACUA028174 | AGAP011989 | 30min PBM_Transcript_7504 | 13.5053686 |
| 24. | XP_321007 | Or77 | ACUA025905 | AGAP002044 | 30min PBM_Transcript_3511 | 20.4188311 |
| 25. | XP_310173 | Or2 | ACUA019491 | AGAP009519 | 30min PBM_Transcript_6920 | 25.7922077 |
| 26. | XP_320128 | Putative GPCR class Orphan Receptor 16 | ACUA001535 | AGAP012427 | 30min PBM_Transcript_5058 | 9.13140271 |
| 27. | XP_308379 | IR75k | ACUA022596 | AGAP007498 | 30min PBM_Transcript_6656 | 9.88007955 |
| 28. | XP_003435724 | IR75I | ACUA008736 | AGAP005466 | 30min PBM_Transcript_5250 | 41.6081841 |
| 29. | EFR28910 | Or23 | ACUA005690 | AGAP007797 | 30min PBM_Transcript_7463 | 19.515343 |
| 30. | XP_314478 | Or44 | ACUA017149 | AGAP010505 | 30hr PBM_Transcript_8297 | 3270.8534 |
| 31. | XP_320541 | Or61 | ACUA019523 | AGAP011991 | 30hr PBM_Transcript_3929 | 16.2275377 |
| 32. | EFR26255 | Putative GPCR class an orphan receptor 21 | ACUA001749 | AGAP005681 | 30hr PBM_Transcript_5866 | 5731.48201 |
| 33. | XP_309588 | Putative GPCR class an orphan receptor 4 | ACUA021279 | AGAP004034 | 30hr PBM_Transcript_8157 | 1696.81659 |
| 34. | XP_320564 | IR76b | ACUA015047 | AGAP011968 | 30hr PBM_Transcript_4542 | 48.3445393 |
| 35. | EFR26426 | IR76a | ACUA001357 | AGAP004923 | 30hr PBM_Transcript_7611 | 21.7380598 |
|  | **Common Receptor Genes expressed in all the experimental conditions.** | | | | |  |
| 36. | ACQ55870 | Or 45 | ACUA019417 | AGAP003053 | 30hr PBM_Transcript_4029 | 19.0208024 |
| 37. | XP_556129 | Gustatory Receptor | ACUA021260 | AGAP005495 | Naive_Transcript_2080 | 7531.73118 |
| 38. | XP_320553 | Or62 | ACUA008089 | AGAP011978 | Naive_Transcript_1886 | 2125.35094 |
| 39. | ABK97614 | Gr24 | ACUA010490 | AGAP001915 | Naive_Transcript_7268 | 661.574914 |
| 40. | XP_312379 | Orco | ACUA003469 | AGAP002560 | Naive_Transcript_972 | 425.790389 |
| 41. | XP_320874 | Or11 | ACUA013073 | AGAP011631 | Naive_Transcript_3225 | 341.520417 |
| 42. | XP_319142 | Gr22 | ACUA004054 | AGAP009999 | Naive_Transcript_1868 | 148.739849 |
| 43. | ACS83758 | Or66 | ACUA000316 | AGAP003310 | Naive_Transcript_5257 | 66.5066074 |
| 44. | XP_312203 | Or28 | ACUA009796 | AGAP002722 | Naive_Transcript_2977 | 47.8300944 |
| 45. | XP_314480 | Or24 | ACUA011915 | AGAP010507 | Naive_Transcript_6099 | 78.4083113 |
| 46. | XP_001688726 | Or33 | ACUA020367 | AGAP005760 | Naive_Transcript_3900 | 25.2802894 |
| 47. | XP_307763 | Gr17 | ACUA019767 | AGAP003255 | Naive_Transcript_7167 | 25.2328592 |
| 48. | XP_319861 | Or29 | ACUA025107 | AGAP009111 | Naive_Transcript_5446 | 53.3175334 |
| 49. | XP_321153 | Or8 | ACUA014383 | AGAP001912 | 30minPBM_Transcript_1795 | 259.150277 |
| 50. | XP_313200 | Or42 | ACUA024848 | AGAP004278 | Naive_Transcript_7008 | 18.1254905 |

**Primer Details:**

| **Serial No.** | **Primer Name and Sequence** |
| --- | --- |
|  | OBP5_Fw: 5’CGGGAACTAAAATGCTACAC 3’  OBP5_Rev: 5’CGTAAGCTATTAACGGTGCT 3’ |
|  | OBP57_Fw: 5’GAAGGATTTGTCAAGCAGTG 3’  OBP57_Rev: 5’GCTTACGTCCTCCTTGTTG 3’ |
|  | SAP_Fw: 5’CAGCAGTACACCACCAAGTA 3’  SAP_Rev: 5’GAGGTAGTTGATCACCTGGA 3’ |
|  | SAP2_Fw: 5’AGGACAAGTACACCACCAAG 3’  SAP2_Rev: 5’GGTAGTTGATCACCTTCTCG 3’ |
|  | OBP1_Fw: 5’ACGAAAAGCTCAAGTGCTAC 3’  OBP1_Rev: 5’GGAAGTAGTGCTTTGGATCA 3’ |
|  | OBP56_Fw: 5’GACGGAAGAAGGAAGTCATC 3’  OBP56_Rev: 5’GCAGTAACCAAAGTGAGAGG 3’ |
|  | OBP58_Fw: 5’CTCGTGTGAAGCAAGATGT 3’  OBP58_Rev: 5’CCAAAGATGTCCTCTGCTAC 3’ |
|  | OBP35_Fw: 5’CTGAGTACGTCCCAAGCTAC 3’  OBP35_Rev: 5’GAGTCTCAGCATTGTCCTTC 3’ |
|  | OBP10_Fw: 5’GGAAGTGAAGGGCTACAAG 3’  OBP10_Rev: 5’ATCAGCTTGGTGAGAAACAC 3’ |
|  | OBP 7_Fw: 5’ GTGCTTGGATGGAACCGTG 3’  OBP 7_Rev: 5’ GGCGGTATCACATTTATCCGG 3’ |
|  | OBP 20_Fw: 5’ CCGTTTGCTTGGGAAAGACA 3’  OBP 20_Rev: 5’GATACCGTCAGCAGCATTCC 3’ |
|  | Gustatory R45_Fw: 5’ TGCTGGCCTCGTTAACAGTA 3’  Gustatory R45_Rev: 5’ CCGTAAATGCTAGCCGGAAG 3’ |
|  | OLF Receptor_Fw: 5’ CATATGGTGTCTTATGCTCTGCT 3’  OLF Receptor_Rev: 5’ TGATGGGTTTCTGGGAACGT 3’ |
|  | Circadian PC_Fw: 5’GGGTTCCTATTTGTGGTCGG3’  Circadian PC_Rev: 5’TCCGTCTTGACTGGAAGCAT3’ |
|  | Or62_Fw: 5’ TGGTATCAACGCAGAGTACA 3’  Or62_Rev: 5’ AGACCGAAGGTGCAGTAGTA 3’ |
|  | Orphan R21_Fw: 5’ GATCCACGATAAGGAGTACG 3’  Orphan R21_Rev: 5’ TCTTCCAGAACGAGTTGAGT 3’ |
|  | Or44_Fw: 5’ CTTCATTTGTCGGTCTTGAT 3’  Or44_Rev: 5’ CCTCAAAGAACTTGCGATAC 3’ |
|  | Adenylate cyclase_Fw: 5’TGGTATCAACGCAGAGTACA 3’  Adenylate cyclase_Rev: 5’ TAACCCTTCGTTTGCAGTAG 3’ |
|  | Or39_Fw: 5’ TTCGATTCACAGAACTCCTT 3’  Or39_Rev: 5’ GCCTTAGCTCTTCGTTTACA 3’ |
|  | IR41c_Fw: 5’ TACGTCATCGAGGGTATGAT 3’  IR41c_Rev: 5’ TAAGCCCAACGTGTCTTATC 3’ |
|  | Uncharacterized Protein: 5’ TTTAGGTTGTTCCTGCAGTC 3’  Uncharacterized Protein: 5’ ACTGATGTGAAGCAGAATCC 3’ |
|  | Or9_Fw: 5’ AGATTGCCTACAACTTCACC 3’  Or9_Rev: 5’ CAGGATCCAGAATATGGTTG 3’ |
|  | Putative OR_Fw: 5’ CTATCTTTGTGCATTTGCTG 3’  Putative OR_Rev: 5’AGATTAAAACGCACAAGAGC 3’ |
|  | OR42_Fw: 5’ TGGTATCAACGCAGAGTACA 3’  OR42_Rev: 5’ GTACAGCTGGACACTTCGAC 3’ |
|  | IR 76b_Fw: 5’ ACATGATCTACGCGGACTAT 3’  IR 76b_Rev: 5’ GATCCTGCAGCTTGTACTTC 3’ |
|  | As_SAP_Fw: 5’ TGGACGAGATCCTCAAGTC 3’  As_SAP_Rev: 5’GTTCTCCGGATCGTACTTCT 3’ |
|  | As_SAP 2_Fw: 5’ AGTACGATGGTGTCGATCTG 3’  As_SAP2_Rev: 5’ ACTTCTTCTGCAGGTTCTCC 3’ |
|  | As_OBP10_Fw: 5’ AAGTGAAGGGATACAAGCTG 3’  As_OBP10_Rev: 5’CGTATGTCACACTGCTTCAC 3’ |
|  | As_OBP20_Fw: 5’ GTTACGTGAACTGCGTGAT 3’  As_OBP20_Rev: 5’ GTTCTTCGAAAGGCACTGA 3’ |
|  | As_OBP7_Fw: 5’ GTCTGTTCGACAAGATCGAC 3’  As_OBP7_Rev: 5’ AACTTGATCACCTCATCGTG 3’ |
|  | As_OBP1_Fw: 5’ GACGAAAAGCTCAAGTGCTA 3’  As_OBP1_Rev: 5’ AGAATCTTACCTTCGGGTCA 3’ |

**Table S6 - Showing major differences between *Anopheles culicifacies* and *Anopheles stephensi,* two major Indian Malaria vectors*:***

| ***Anopheles culicifacies*** | ***Anopheles stephensi*** |
| --- | --- |
| 1. ***Anopheles culicifacies*** contributes to about 60-65% of all malaria cases in India from rural to peri-urban areas and is widely distributed throughout the country. 2. **Species complexity**: *An. culicifacies* sp. comprised five sibling species provisionally designated as species A, B, C, D and E. 3. **Host Preference:** All the members of *An. culicifacies* are predominantly zoophilic except species E and rest indoor mainly in cattle sheds. Besides its low Anthropophagy, it acts as a major malaria vector due to the fact that it is found in high density. 4. **Breeding preference:** The preferred breeding sites are streams rice fields, seepage water borrow pits, irrigation channels, rain water collection etc. 5. **Biting Behaviour:** About 70–90% of *An. culicifacies* population caught during the whole night was found to feed prior to midnight during the months of January to April. Bimodal activity was seen during June and July indicating a further shift towards the second and third quarters of the night. During August–September, most biting takes place in the latter part of the night. | 1. ***Anopheles steph****ensi* is an important vector of malaria in urban areas.      1. **Species complexity**: *An. stephensi* Liston 1901lack sibling species complex, but exists as two forms, the type form and the variety *mysorensis*, which are distinguished by differences in the egg length and width and by the number of ridges on the egg float. 2. **Host Preferences:** In cities, it is the main malaria vector and has shown an increased tendency to feed on man than on cattle unlike in rural areas. 3. **Breeding preference:** *An. stephensi* is found predominantly in clear water, except in some polluted, blocked cemented drains with grass growth in the non-riverine zone near Delhi 4. ***Biting Behaviour:*** Biting occurs mostly before midnight and maximum activity was observed in the first quarter of the night (1800–2100 hrs). Biting activity has been observed till third and fourth quarters of the night, though at a low rate. *An. stephensi* was active throughout the night and 39% population fed before midnight. |
